# Ambient direct arylation synthesis of thienothiophene based copolymers with mixed alkoxy and oligoether side chains

**DOI:** 10.1039/d6sc01881e

**Published:** 2026-05-26

**Authors:** Di Zhu, Judith Pons i Tarrés, Joost Kimpel, Meghna Jha, Mariavittoria Craighero, Jesika Asatryan, Alberto Peinador Veiga, Zesheng Liu, Tania Cecilia Hidalgo, Megan M. Westwood, Mats Fahlman, Jaime Martín, Alexander Giovannitti, Christian Müller

**Affiliations:** a Department of Chemistry and Chemical Engineering, Chalmers University of Technology 41296 Gothenburg Sweden christian.muller@chalmers.se; b Universidade da Coruña, Campus Industrial de Ferrol, CITENI Esteiro Ferrol Spain; c Laboratory of Organic Electronics, Department of Science and Technology, Linköping University Norrköping Sweden

## Abstract

Conjugated polymers with both oligoether and alkoxy side chains are an emerging type of organic mixed ionic-electronic conductor. Here, direct arylation polymerization is introduced as a viable synthetic route for such materials. The composition and molecular weight of the synthesized thienothiophene-based random copolymers are determined with high-temperature ^1^H NMR spectroscopy. Chemical and electrochemical doping of the copolymers reveal that the introduction of a minor fraction of alkoxy side chains results in materials with a highly promising set of electrical and mechanical properties. A copolymer with an alkoxy side-chain fraction of 12% features an electrical conductivity of 390 S cm^−1^ and a charge-carrier mobility of more than 3 cm^2^ V^−1^ s^−1^. Organic electrochemical transistors with a NaCl aqueous electrolyte feature a high figure of merit of 1313 F cm^−1^ V^−1^ s^−1^ thanks to a high volumetric capacitance of 417 F cm^−3^. Evidently, the introduction of a minor fraction of alkoxy side chains is a viable route for improving the electrical properties of organic mixed ionic-electronic conductors.

## Abstract 2

Thienothiophene-based copolymers bearing mixed oligoether and alkoxy side chains were synthesized *via* ambient direct arylation polymerization. Introducing a minor alkoxy fraction helps improve the electrical properties of organic mixed conductors.

## Graphical abstract

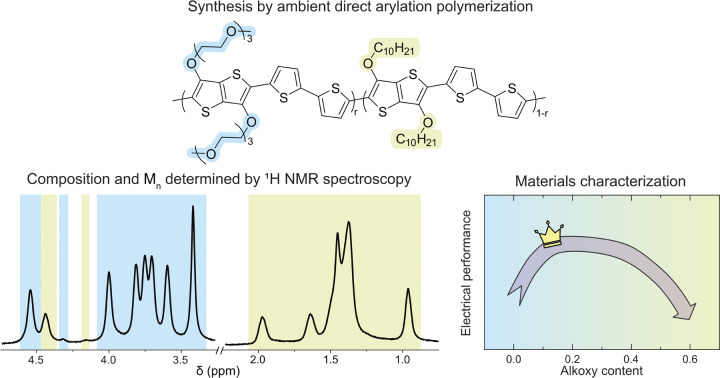

## Introduction

Conjugated polymers receive considerable attention for a wide range of electronic applications from energy harvesting and storage to bioelectronics because they combine promising electrical and mechanical properties with the potential for biocompatibility and low-cost processing.^1–6^ The electrical properties of conjugated polymers can be tuned through chemical and electrochemical doping, which involves mass transport of polar dopant molecules and/or counterions.

Oligoether side chains are widely used to ensure compatibility with polar dopants^7–9^ and to facilitate ion ingression.^10^ For example, polythiophenes with oligoether side chains possess a higher degree of stability compared to poly(3-hexylthiophene) (P3HT) when oxidized with the dopant 2,3,5,6-tetrafluoro-tetracyanoquinodimethane (F_4_TCNQ) because the sublimation of the dopant is suppressed.^8,9^ Likewise, a comparison of thienothiophene based polymers with alkoxy or oligoether side chains has shown that the latter facilitate ion ingression and thus bulk doping during electrochemical oxidation.^11^ Moreover, polymers with oligoether side chains feature a higher dielectric constant than their alkyl counterparts, which can benefit charge dissociation and transport.^4,12^

The choice of side chain strongly influences the type and extent of solid-state packing of polymer films, which in turn strongly impacts charge transport. A combination of scanning tunnelling microscopy (STM) and molecular dynamics (MD) simulations has revealed that alkyl side chains tend to adopt an all-trans configuration compared to more twisted oligoether side chains, resulting in more regular packing of side chains in case of the former.^13^ Likewise, grazing-incidence wide-angle X-ray scattering (GIWAXS) has shown that films composed of polythiophenes or thienothiophene based polymers with triethylene glycol side chains feature poor lamellar but strong π–π stacking whereas polymers with alkyl or alkoxy side chains tend to feature more ordered lamellar domains in addition to π–π stacking.^11,14^ The use of longer tetra- or hexaethylene glycol side chains also suppresses π–π stacking of polythiophenes and thienothiophene based polymers.^7,15,16^

Therefore, it can be beneficial to create materials that comprise both alkyl or alkoxy side chains and oligoether side chains. This has been achieved by mixing two polymers that feature alkoxy and oligoether side chains, respectively.^17^ Alternatively, to avoid micrometer-scale phase separation, polymers have been synthesized that feature both moieties in the same covalently-linked system. Many studies have described p- and n-type polymers with hybrid side chains composed of a mono- or oligoether moiety covalently linked to an alkyl spacer, which can improve polymer-substrate adhesion and modulate swelling, resulting in organic electrochemical transistors (OECTs) with enhanced performance and stability.^18–25^ However, hybrid side chains must be assembled from discrete alkyl and (oligo)ether building blocks, typically through multi-step syntheses, resulting in a relatively high synthetic complexity compared to pure alkyl, alkoxy and oligoether side chains, whose precursors are readily available.

An alternative approach involves the preparation of random copolymers that combine two types of monomers with alkyl/alkoxy and oligoether side chains, respectively.^26,27^ Often, the two monomers can be prepared by a similar reaction scheme using the same conjugated precursor in combination with either an alkyl/alkoxy or an oligoether moiety, which, ideally, are commercially available. This significantly simplifies the synthesis workflow, improving the overall scalability of the target polymer compared to materials comprising hybrid side chains. For example, Siemons *et al.* prepared random copolymers with an all-thiophene backbone and different alkoxy:oligoether side-chain ratios by Stille coupling using the same catalyst loading and reaction conditions.^26^ Stille coupling polymerization was also used for the synthesis of naphthalenediimide- and isoindigo-based random copolymers with mixed side chains.^27,28^ A disadvantage of Stille coupling is the need for toxic organotin compounds that require an additional functionalization step, which leads to a relatively high synthetic complexity index (SCI).

**Fig. 1:**
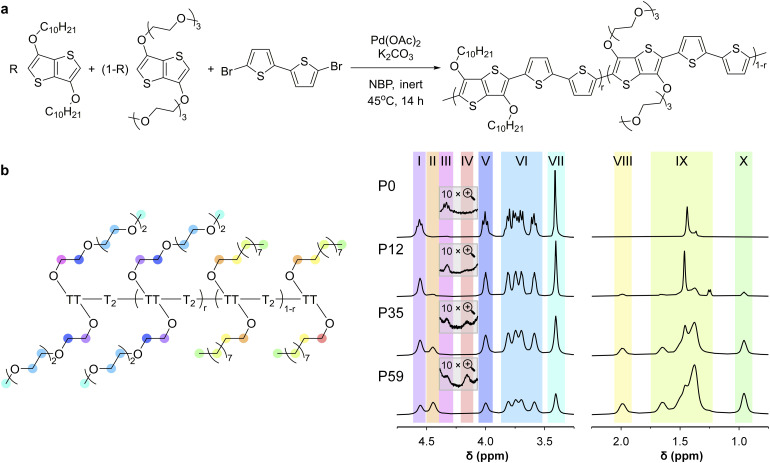
Synthesis of random copolymers p[(g_3_TT-T2)-*ran*-(a_10_TT-T2)]. (a) General synthesis scheme and (b) high-temperature ^1^H NMR spectra of P0–P59 recorded at 120 °C in C_2_D_2_Cl_4_; colours indicate the protons associated with various NMR signals; P0 corresponds to entry 9 described in ref. 33.

One of the most widely studied families of p-type conjugated polymers with oligoether side chains combines a thienothiophene and a bithiophene comonomer.^11,13,29^ Positioning of the oligoether side chains on the thienothiophene moiety has only been reported recently.^30–32^ This side-chain substitution pattern is attractive because monomers such as 3,6-bis(triethylene glycol monomethyl ether)thieno[3,2-*b*]thiophene (g_3_TT) are ideally suited for direct arylation polymerization (DAP).^31,32^ DAP is advantageous because, in contrast to Stille coupling, no toxic monomers are required. Recently, our group has reported that g_3_TT can be copolymerized at room temperature in an open-flask reaction with various aryl dibromides such as 5,5′-dibromo-2,2′-bithiophene (Br-T2-Br).^33^ The resulting ambient direct arylation polymerization (ADAP) is attractive because it facilitates a very low SCI and yields copolymers such as p(g_3_TT-T2) (see Fig. 1a for chemical structure) with a promising OECT performance.^33^ Neither DAP nor ADAP have been used for the synthesis of random copolymers with mixed alkoxy/alkyl and oligoether side chains.

Here, we report the synthesis of the monomer 3,6-bis(decyloxy)thieno[3,2-*b*]thiophene (a_10_TT) and its random copolymerization together with g_3_TT at different feed ratios, which is the first use of DAP for the preparation of mixed side-chain random copolymers. The molecular weight and alkoxy side-chain fraction of the resulting random copolymers were determined with high-temperature ^1^H NMR. Copolymers with an alkoxy side-chain fraction of up to 35% featured a number-average molecular weight of 13 kg mol^−1^ as well as promising electrical properties upon chemical and electrochemical oxidation that favourably compare with those of the all-oligoether substituted copolymer. In particular, the copolymer with an alkoxy side-chain fraction of 12% exhibits a conductivity of about 390 S cm^−1^ upon chemical doping with F_4_TCNQ, along with an OECT figure of merit of 1313 F cm^−1^ V^−1^ s^−1^ when using a NaCl aqueous electrolyte.

## Results and discussion

To facilitate the synthesis of random copolymers with alkoxy and oligoether side chains, we prepared a thienothiophene monomer by Ullmann-type coupling of decan-1-ol to 3,6-dibromothieno[3,2-*b*]thiophene, achieving a yield of 45% after recrystallization from hexane, which is comparable to the yields reported for g_3_TT^31^ (see SI Fig. S1–S7). The resulting a_10_TT monomer featured decyloxy side chains (–OC_10_H_21_), which contain the same number of C + O atoms as the triethylene glycol monomethyl ether chains of g_3_TT, and thus they have a similar length. The single crystal structure of a_10_TT (Fig. S7) confirms the all-trans configuration preference of decyloxy side chains in the solid state, resulting in a more linear conformation compared to the twisted triethylene glycol monomethyl ether chains of g_3_TT.^31,34^

For the preparation of random copolymers comprising both a_10_TT and g_3_TT, a solvent system was needed that dissolved both monomers as well as growing chains irrespective of the monomer ratio. Initially, we performed ADAP of a_10_TT and 5,5′-dibromo-2,2′-bithiophene (Br-T2-Br) in chlorobenzene, which had been used in previous studies that explored Stille coupling of random copolymers with mixed side chains.^27,28^ However, we did not observe any colour change as time progressed, which indicated that no polymerization had occurred. This is consistent with a previous report where we found that the bimetallic Pd^II^/Pd^0^ catalytic system, which underpins ADAP based on Pd(OAc)_2_, requires an amide solvent.^33^ Moreover, chlorobenzene has a low polarity as evidenced by a low Reichardt scale of polarity value of *E*^N^_T_ = 0.188,^35^ which would be detrimental for dissolving g_3_TT and g_3_TT-rich copolymers. A solvent system with sufficient amide functionality and a polarity in between chlorobenzene and DMAc would be more suited, such as a 1 : 1 mixture. Therefore, we conducted the synthesis of p(a_10_TT-T2) in a 1 : 1 chlorobenzene : DMAc solvent mixture. The resulting yield and molecular weight of the fraction extracted from CHCl_3_ was 45% and 3 kg mol^−1^.

Previously, batch reactions involving ADAP of p(g_3_TT-T2) were carried out in dimethylacetamide (DMAc) or *N*-butyl-2-pyrrolidone (NBP). NBP, with a value of *E*^N^_T_ = 0.323 is less polar than DMAc with *E*^N^_T_ = 0.377,^35,36^ which can be expected to lead to a higher compatibility with less polar alkoxy functionalized content. Hence, NBP was deemed more suitable for the synthesis of copolymers with mixed side chains. We observed that ADAP of equimolar amounts of a_10_TT and g_3_TT reacting with Br-T2-Br readily occurred. Therefore, NBP, being non-reprotoxic, non-mutagenic and inherently biodegradable,^36^ was used as the solvent for synthesizing the targeted random copolymer series with mixed alkoxy and oligoether side chains. Compared to previously reported conditions for ADAP,^33^ we slightly lowered the monomer loading concentration from 100 to 50 mM and elevated the reaction temperature from 25 to 45 °C, while keeping the catalyst loading at 5 mol% (Fig. 1a). The purpose was to improve the solubility of the products while keeping the polymerization at a controllable rate, which allowed us to carefully select the termination point. In addition to optimizing the solvent system, we carried out the polymerizations under inert atmosphere to prevent air-oxidation and subsequent precipitation of the yielding copolymers.

The polymerization progress was monitored by observing colour changes as well as precipitation of aliquots of the reaction mixture in *n*-hexanes, methanol, ethyl acetate and chloroform. Once aliquots precipitated in the former three solvents while being soluble in chloroform at 40 °C, the reaction mixture was precipitated in methanol to quench the polymerization. After purification by precipitation in methanol followed by Soxhlet extraction with isopropanol, ethyl acetate and chloroform, the chloroform-soluble fraction of each polymer was collected and used for further analysis. Polymerizations with a lower a_10_TT feed molar composition (*R* ≤ 0.3) achieved yields of 40 to 70%, while higher a_10_TT equivalence (*R* ≥ 0.5) led to yields of 30 to 50% (Table S1).

**Fig. 2:**
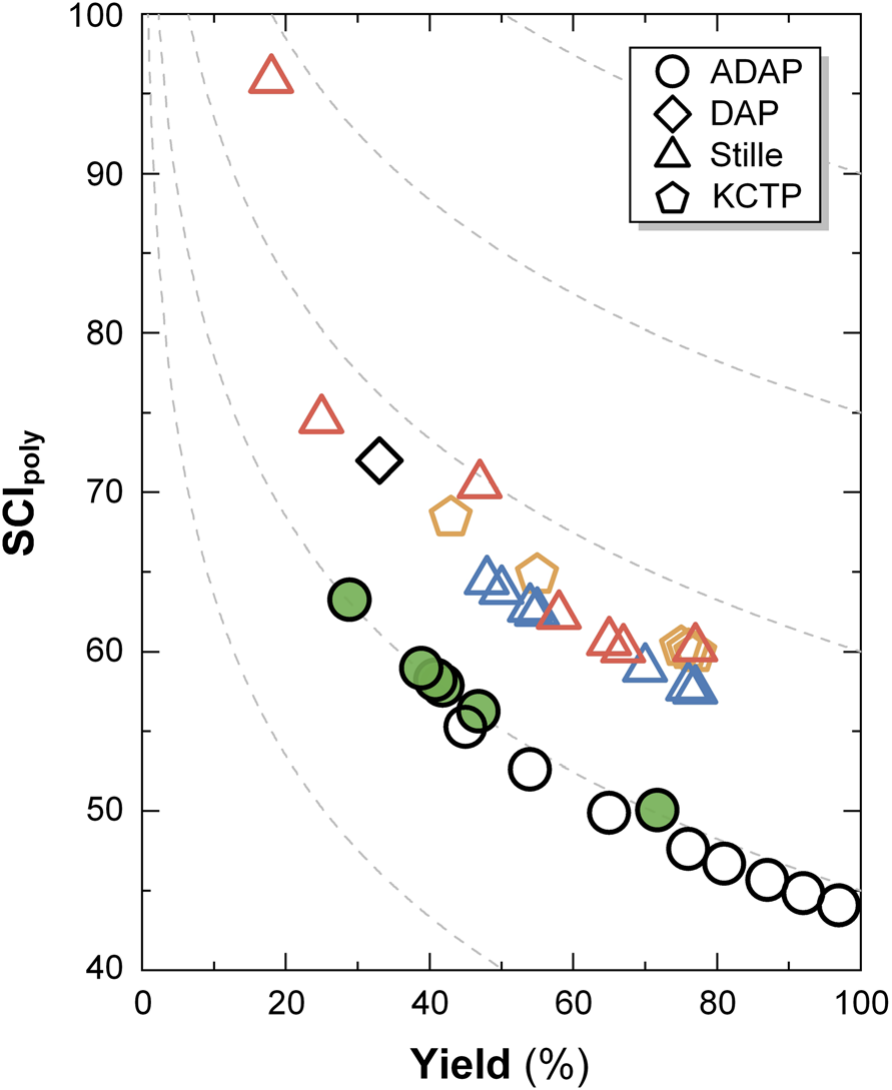
Synthetic complexity index (SCI_poly_) of polymerizations. SCI_poly_*vs.* yield after workup of the synthesized p[(g_3_TT-T2)-*ran*-(a_10_TT-T2)] random copolymers (filled circles), p(g_3_TT-T2) synthesized by ADAP (open circles), previously reported TT-T2 copolymers with oligoether and mixed side chains synthesized by Stille coupling (red triangles) and DAP (diamonds), NDI-T2 copolymers with oligoether, alkoxy and mixed side chains synthesized by Stille coupling (blue triangles), and statistical thiophene copolymers with mixed side chains synthesized by KCTP (orange pentagons).^11,17,26,27,31,33,37,38^ The grey dashed lines are contour lines that illustrate how SCI_poly_ varies with yield for polymerizations having an equal number of synthetic steps, number of purification operations and environmental hazards.

The SCI of the polymerization step, SCI_poly_, of the p[(g_3_TT-T2)-*ran*-(a_10_TT-T2)] random copolymers was calculated (Fig. 2). SCI_poly_ evaluates a polymerization reaction with regard to multiple aspects, including yield, the number of synthetic steps, the number of purification operations, the number of columns and environmental hazards (Table S3). SCI_poly_ values range from 63 to 50, depending on the yield of the polymerization, and compare favourably with other mixed side-chain TT-T2, NDI-T2 and polythiophene based copolymers made by DAP, Stille coupling or Kumada catalyst transfer polymerization (KCTP).

High-temperature ^1^H NMR spectroscopy was used to analyse the chemical structures of the synthesized copolymers, including the composition and molecular weight of the random copolymers and p(a_10_TT-T2). (Fig. S8–S13). ^1^H NMR spectra of the monomers and previously reported conjugated polymers with oligoether and/or alkoxy side chains allowed us to assign the various NMR signals (Fig. 1b).^11,17,26–28,31,33^

Comparison of the NMR spectra recorded for the copolymers and p(g_3_TT-T2) revealed an increase in the intensity of the peaks at 2.13 to 0.88 ppm (alkoxy region) and a decrease of those at 4.66 to 4.00 ppm (oligoether region) with increasing alkoxy:oligoether monomer feed fraction of a_10_TT (Table 1). This indicates that the feed fraction determines the composition of the synthesized random copolymers.

**Table 1:** Stoichiometry and molecular weight. Monomer fraction of a_10_TT *R* = a_10_TT/(a_10_TT + g_3_TT) prior to polymerization, fraction *r*_CH_3__ of a_10_TT units in the polymer, relative difference *δr*_CH_3__ = (*r*_CH_3__ − *R*)/*R* and number-average molecular weight 
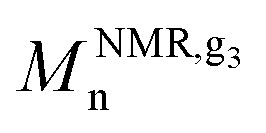
 determined by NMR end-group analysis

Polymer	*R* (−)	*r*_CH_3__ (−)	*δr*_CH_3__ (%)	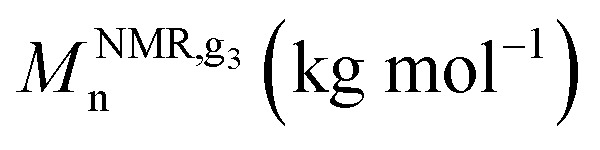
P0	0	0	—	14^a^
P12	0.10	0.12 ± 0.004	20 ± 4	13 ± 1
P35	0.30	0.35 ± 0.002	17 ± 1	13 ± 2
P59	0.50	0.59 ± 0.002	18 ± 1	11 ± 1

a From entry 9 in ref. 33; errors represent the uncertainty estimated by considering the signal-to-noise ratios of individual NMR signals.

**Fig. 3:**
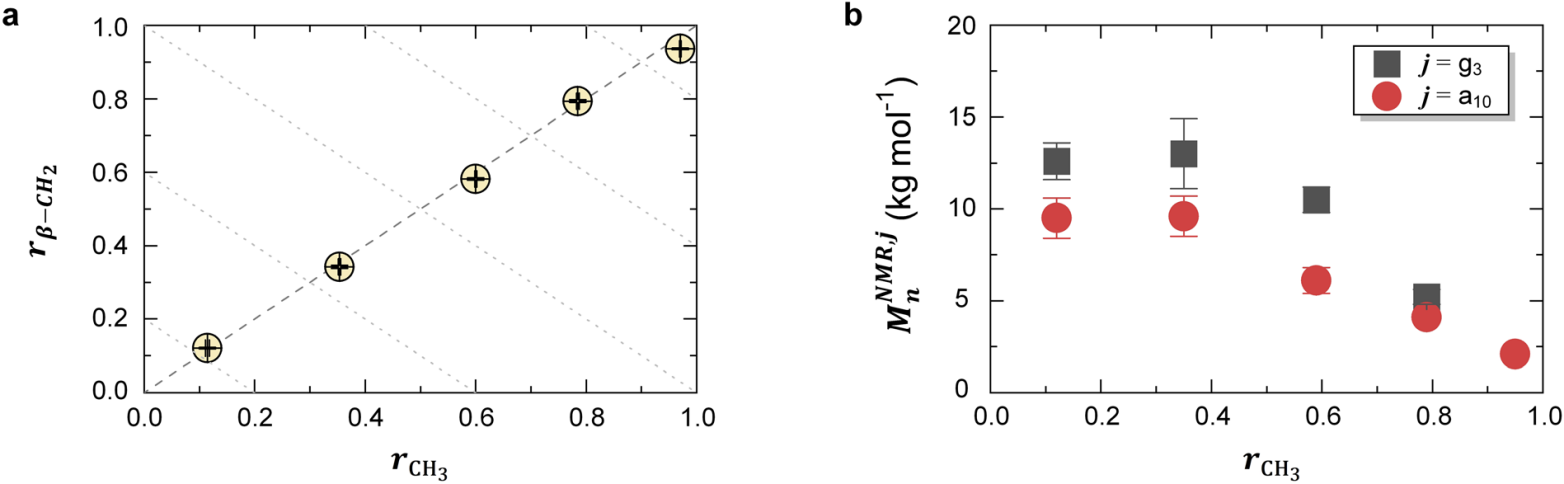
NMR analysis of chemical structures of the random copolymers. (a) Fraction of TT units with alkoxy side chains, *r*_CH_3__ and *r*_β-CH_2__, calculated by comparing the CH_3_ or β-CH_2_ signals according to eqn (1); (b) *M*^NMR,j^_n_ calculated by comparing the integrals of the signals associated with α-CH_2_ from the main-chain *vs.* terminal oligoether side chains (grey squares) and main-chain *vs.* terminal alkoxy side chains (red circles) according to eqn (2) and (3); error bars represent the uncertainty estimated by considering the signal-to-noise ratios of individual NMR signals.

To quantify the composition of each synthesized copolymer, we chose to compare the CH_3_ signals of both types of side chains because they feature well-defined Lorentzian shapes and least overlap with other signals. Compared to CH_2_ signals, the extra proton imparts the CH_3_ peak with a stronger intensity and a significantly better signal-to-noise ratio. Only the signal at about 1.38 ppm has a higher intensity due to overlap with the water peak and other CH_2_ signals. The fraction of TT units with alkoxy side chains, *i.e.* the alkoxy side-chain fraction, was calculated according to:1
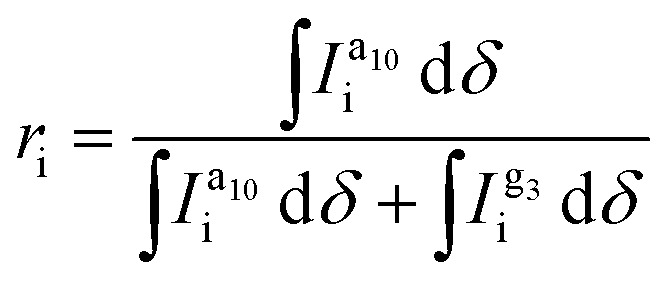
where i denotes the CH_3_ or β-CH_2_ protons of alkoxy and oligoether side chains, 
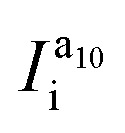
 is the alkoxy signal intensity (regions X or VIII in Fig. 1b) and 
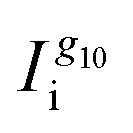
 is the oligoether signal intensity (regions VII or V in Fig. 1b). Comparison of the CH_3_ or β-CH_2_ signals yields similar values for the compositions of the random copolymers (Fig. 3a), which are generally in good agreement with the monomer feed fractions (Table 1). There is a preference for the incorporation of alkoxy monomers over oligoether monomers during the polymerization, as evidenced by an about 20% relative difference between the fraction of a_10_TT units and the monomer feed fraction (Table 1). It can be concluded that the alkoxy bearing a_10_TT monomers exhibit a higher reactivity under the reported reaction conditions (Fig. 1a). We named the random copolymers according to the fraction of TT units with alkoxy side chains. For example, copolymer P12 has a a_10_TT to g_3_TT molar ratio of 12 : 88 since *r*_CH_3__ = 12%.

NMR end-group analysis was used to determine the number-average molecular weight. We assumed that each polymer chain is terminated by either a TT or T2 unit with equal probability, with the former bearing either decyloxy or triethylene glycol side chains with a probability equal to *r*_CH_3__ (see eqn (1)). The number-average molecular weight *M*^NMR,j^_n_ was obtained according to (see SI for details):2
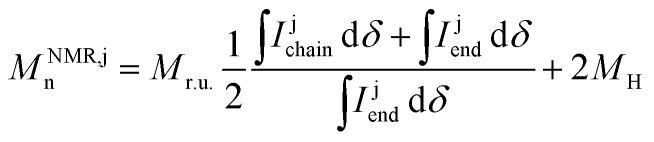
where j = g_3_ or a_10_ denotes signals associated with the α-CH_2_ protons of oligoether or alkoxy side chains, respectively, *M*_r.u._ is the copolymer-dependent average molecular weight of a TT-T2 repeat unit (see SI), *I*^j^_chain_ is the intensity of the signal associated with α-CH_2_ from the side chains of main-chain TT units (region I or II in Fig. 1b), *I*^j^_end_ is the intensity of the signal associated with α-CH_2_ from the side chains of terminal TT units (region III or IV for g_3_ or a_10_, respectively, in Fig. 1b) and *M*_H_ is the molecular weight of a hydrogen at the end of the polymer chain.

ADAP results in TT–TT homocoupling, with typical values of *n*_homo_ = 2 to 7% in case of p(g_3_TT-T2).^33^ We can calculate the error in *M*^NMR,j^_n_ caused by homocoupling according to:

3 Δ*M*^NMR^_n_ [homo] = *M*_T2_·*n*_homo_·*X*^NMR,j^_n_

where *n*_homo_ is the concentration of homocouplings and *M*_T2_ is the moelcular weight of a T2 unit. Using the highest reported value, *i.e. n*_homo_ = 7% for p(g_3_TT-T2),^33^ an upper bound for the error due to homocoupling of Δ*M*^NMR^_n_ [homo] < 1 kg mol^−1^ is obtained. A general trend is that 
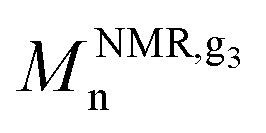
 is always higher than 
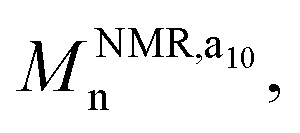
 which is because 
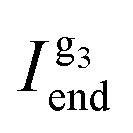
 and 
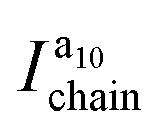
 partially overlap and hence the automated oblique integration causes an underestimation of 
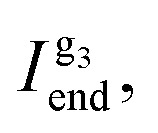
 which results in a higher value for 
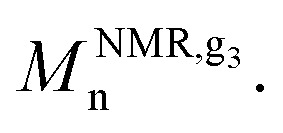
 Copolymers with an alkoxy side-chain fraction of *r* ≤ 0.59 featured a 
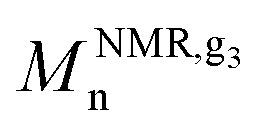
 ≥ 10 kg mol^−1^ (*cf.*Fig. 3b; see Fig. S14 and Table S2 for size exclusion chromatography (SEC) measurements). Instead, copolymers with *r* ≥ 0.79 are oligomeric with an *M*^NMR^_n_ ≤ 5 kg mol^−1^. The molecular weight that can be achieved depends on the solubility of the growing polymer chain in addition to the monomer ratio. The polymerization proceeds until aggregation hinders further chain growth, which suggests that the lower *M*^NMR,j^_n_ of alkoxy side-chain rich copolymers arises because of their inferior solubility in the polar reaction medium NBP compared to oligoether side-chain rich copolymers. A decrease in molecular weight has also been reported for NDI-T2 copolymers with mixed side chains,^27^ albeit with increasing oligoether side-chain content since the reaction was carried out in chlorobenzene, a less polar solvent, which instead promotes solubility of alkoxy side-chain rich copolymers. Note that molecular weight characterization in this work and for the mentioned NDI-T2 copolymers was performed only for the chloroform-soluble fractions from Soxhlet extraction. Hence, the reported values are not representative for the crude products. Given that the molecular weight of conjugated polymers strongly influences their physico-chemical and electrical properties we chose to focus our attention on copolymers with *r* ≤ 0.59 during the remainder of this study since they possess comparable molecular weights above 10 kg mol^−1^.

The CH_3_ signals of oligoether side chains (regions VII in Fig. 1b and peaks at 3.40 ppm in Fig. S8–S13) broaden from P12 to P59 and become narrower from P59 to P97. The observed broadening may originate from increased sequence disorder as the alkoxy and oligoether side-chain contents reach a comparable level, resulting in different local chemical environments around each repeat unit. Regardless, it is unlikely that the distribution of the alkoxy side-chain content within the synthesized copolymers is perfectly random since the a_10_TT and g_3_TT monomers appear to feature slightly different reactivities (*cf.*Table 1). However, X-ray scattering and cyclic voltammetry (CV) measurements support a predominantly random copolymer structure, as discussed below.

**Fig. 4:**
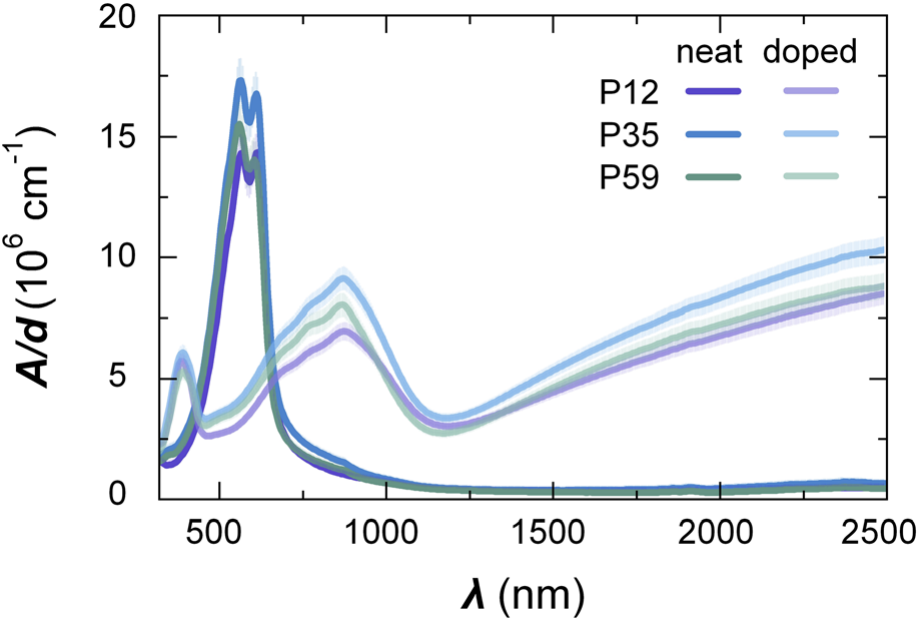
UV-Vis-NIR absorption spectroscopy. Thin-film UV-Vis-NIR absorption spectra of P12–P59 neat films (dark lines) and films sequentially doped with F_4_TCNQ (bright lines) showing the absorbance *A* normalized by the film thickness *d*; the shaded areas indicate the uncertainty that arises from the error in *d*.

Ultraviolet photoelectron spectroscopy (UPS; Fig. S15) and cyclic voltammetry in NaCl aqueous electrolyte (CV; Fig. S16–S18) of neat films revealed that the ionization energy increases with alkoxy side-chain content from IE^CV^ = 4.43 eV in case of P0 to 4.82 eV for P59 (Table 2). Furthermore, each polymer features only one oxidation onset, which is consistent with a mostly random distribution of a_10_TT and g_3_TT monomers along the polymer chain. UV-Vis-NIR absorption spectroscopy suggested that the optical band gaps of P12–P59 are similar, *i.e.* Δ*E*^opt^_g_ ≈ 1.8 eV (Fig. 4 and Table 2). UV-Vis-NIR spectra of pristine P12–P59 feature well-resolved absorption bands in the visible light region at 560 nm and at 610 nm. We assign the latter to the presence of aggregates in analogy to P3HT.^39^ These two absorption bands are of similar intensity in case of P12 while in case of P35 and P59 the absorption at 560 nm is stronger.

**Table 2:** Ionization energy and bandgap. Oxidation onset *E*^CV^_ox_*vs.* Ag/AgCl from cyclic voltammetry (CV), ionization energy from cyclic voltammetry (CV) and ultraviolet photoelectron spectroscopy (UPS), IE^CV^=*E*^CV^_ox_ + 4.4 eV^41^ and IE^UPS^, and optical band gap Δ*E*^opt^_g_ from UV-Vis-NIR spectroscopy

Polymer	*E* ^CV^_ox_ (eV)	IE^CV^ (eV)	IE^UPS^ (eV)	Δ*E*^opt^_g_ (eV)
P0^a^	0.03	4.43	—	1.80
P12	0.22	4.62	4.77	1.79
P35	0.32	4.72	4.79	1.81
P59	0.42	4.82	—	1.83

a Extracted from entry 9 in ref. 33.

Sequential doping of P12–P59 thin films with 5 mM of F_4_TCNQ dissolved in a degassed solvent mixture of 5 vol% ethyl acetate in acetonitrile resulted in complete bleaching of the neat polymer absorption along with the appearance of absorption peaks associated with the F_4_TCNQ anion at ≈770 and ≈870 nm^15^ and polaronic peaks in the NIR region (Fig. 4). In a previous study, we used X-ray photoelectron spectroscopy (XPS) to determine the charge-carrier concentration *N* of chemically doped p(g_3_TT-T2) thin films.^40^ XPS of strongly doped films, whose UV-Vis-NIR spectra also indicate complete bleaching of the neat polymer absorption, yielded a value of *N* ≈ 7 × 10^20^ cm^−3^. Hence, we argue that the here investigated films of P12–P59 are doped to a similar extent.^40^

**Fig. 5:**
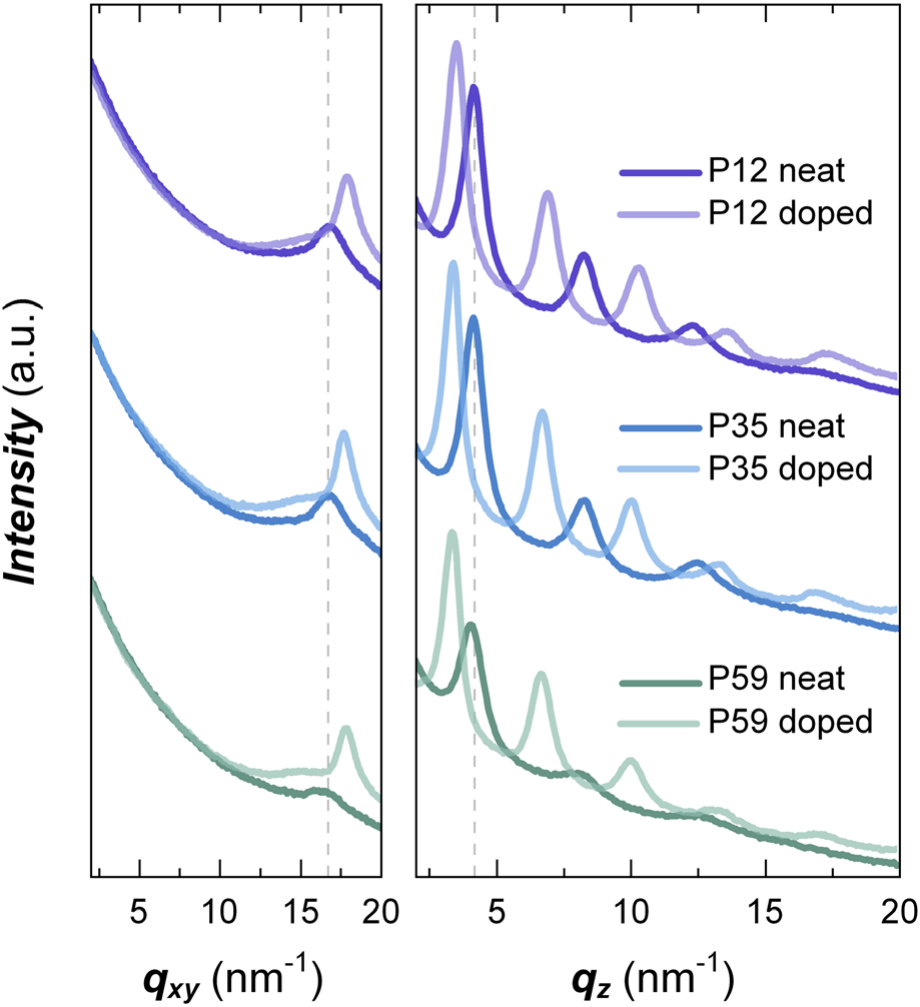
Grazing incidence X-ray scattering (GIWAXS) line cuts. In-plane (left panel) and out-of-plane line cuts (right panel) of GIWAXS patterns of neat films (dark lines) and films sequentially doped with F_4_TCNQ (bright lines).

Grazing incidence wide-angle X-ray scattering (GIWAXS) was performed to investigate how the alkoxy side-chain fraction influences the solid-state nanostructure of neat and doped films (see Fig. 5 for in-plane and out-of-plane line–cut profiles and Fig. S22 for GIWAXS patterns). GIWAXS line–cut profiles of pristine P12–P59 films feature three out-of-plane diffraction peaks indicative of lamellar ordering with *q*_100_ ≈ 4.1 nm^−1^ and an in-plane diffraction peak characteristic of π–π stacking with *q*_010_ ≈ 16.7 nm^−1^, suggesting a predominant edge-on orientation for all three copolymers. Strikingly, we observe only one set of lamellar and π–π stacking peaks for all copolymers from P12 to P59, indicating the presence of a single type of ordered domain, which is consistent with a random instead of a blocky copolymer architecture. Like p(g_3_TT-T2),^42^ GIWAXS patterns of both P12 and P35 exhibit strong reflections, which indicates a high degree of order despite introducing a significant amount of comonomers with alkoxy side chains, which agrees with previous studies of other copolymers.^17,28^ Instead, P59 with a higher alkoxy side-chain fraction shows weaker diffraction peaks, which was also observed in case of NDI-based copolymers.^27^ The predominantly random distribution of a_10_TT and g_3_TT units implies that in case of P59 only few regular segments exist, and as a result the material features a low degree of structural order.

Sequential doping of P12–P59 films with F_4_TCNQ results in a notable improvement in the degree of order, as indicated by the increase in the intensity of diffraction peaks at *q*_100_ ≈ 3.5 nm^−1^ and *q*_010_ ≈ 18.0 nm^−1^. In addition, higher-order diffraction peaks associated with lamellar stacking appear at *q*_400_ ≈ 13.6 nm^−1^ and *q*_500_ ≈ 17.2 nm^−1^. The lower *q*_100_ value compared to that of the neat films reveals the expansion of the lamellar spacing, which we explain with intercalation of counterions between the side chains, in agreement with other studies that investigate chemical doping of thienothiophene based copolymers including P0.^40,42^

**Fig. 6:**
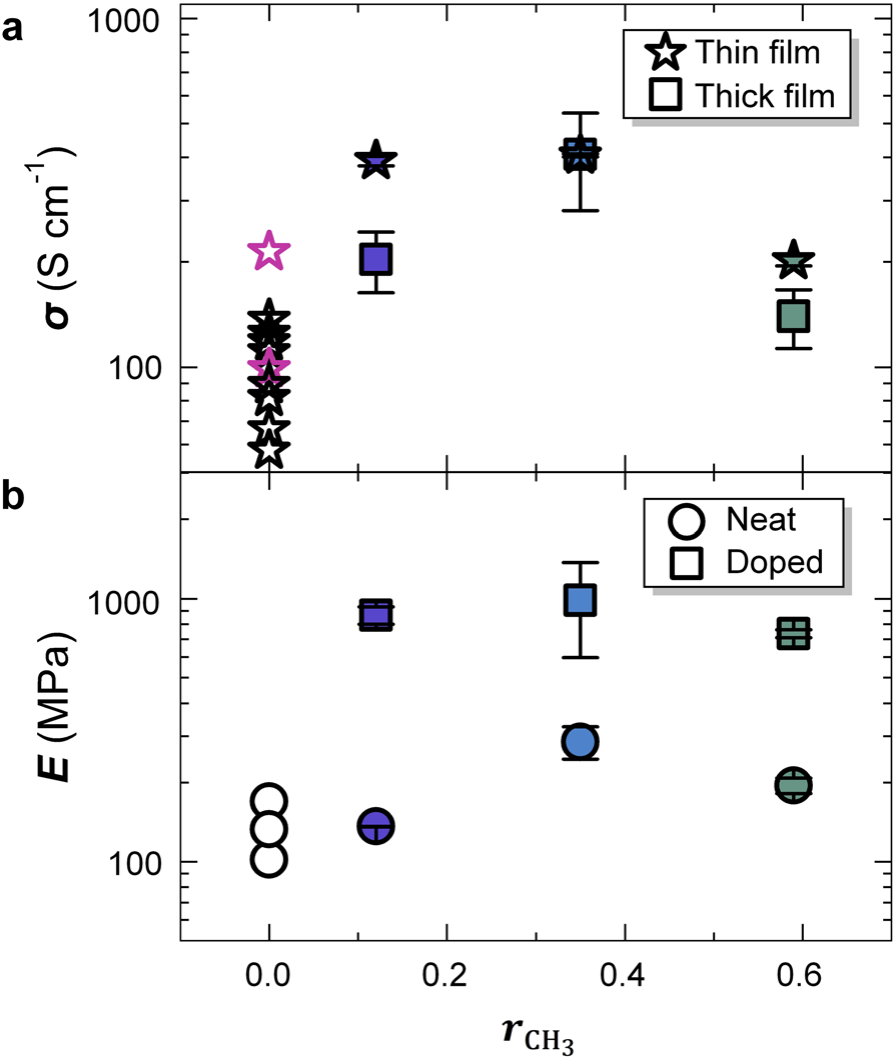
Electrical and mechanical properties. (a) Electrical conductivity *σ* of F_4_TCNQ-doped 25–40 nm thin (stars) and 2–8 µm thick films (squares) doped with F_4_TCNQ; and (b) elastic modulus *E* of 2–8 µm thick films *vs.* the alkoxy side-chain fraction *r*_CH_3__ before (circle) and after doping with F_4_TCNQ (squares) measured as part of this study (filled symbols) or taken from literature (open symbols);^40,45^*σ* values are obtained by measuring one sample and error bars reflect the uncertainty in resistance and thickness while in case of *E* the mean and standard deviation of 9 nanoindentation creep measurements of the same sample are shown.

We measured the electrical conductivity *σ*_thin_ of 25 to 40 nm thin F_4_TCNQ-doped films to investigate the effect of alkoxy side chains on charge transport. All three copolymers bearing both oligoether and alkoxy side chains displayed a high *σ*_thin_ above 200 S cm^−1^, with P35 showing the highest value of *σ*_thin_ = (405 ± 4) S cm^−1^ (Fig. 6a and Table S5). In contrast, for F_4_TCNQ-doped P0 and other ADAP-synthesized p(g_3_TT-T2) batches values ranging from 58 to 214 S cm^−1^ have been reported (Fig. 6a).^40^ Thickness normalized UV-Vis-NIR spectra of P12–P59 revealed that these three copolymers have similar oxidation levels (Fig. 4), resulting in a charge-carrier mobility *µ* = *σ*_thin_/*eN* ≈ 3.6 cm^2^ V^−1^ s^−1^ for P12 and P35 while P59 films have a lower value of 1.8 cm^2^ V^−1^ s^−1^. Strongly oxidized p(g_3_TT-T2) films, *e.g.* films sequentially doped with F_4_TCNQ, feature values of *µ* ≈ 1.9 cm^2^ V^−1^ s^−1^.^42^ Therefore, the higher value of *σ*_thin_ in case of P12 and P35 thin films compared to P0 can be attributed to a higher charge-carrier mobility.

Conjugated polymer films with thicknesses exceeding one micrometer are relevant for devices such as thermoelectric generators where bulk transport, electrochemical activity, and mechanical robustness are required.^43,44^ Hence, we also prepared 2–8 µm thick films by drop casting and again carried out sequential doping with F_4_TCNQ. Doped P35 films with a thickness of 1.8 µm featured an electrical conductivity *σ*_thick_ = (408 ± 127) S cm^−1^ comparable to that of thin films (Fig. 6a and Table S5). Instead, in case of P12 and P59 films with thicknesses of 5.6 and 8.1 µm we observed significantly lower values compared to thin films.

Nanoindentation creep experiments (see Experimental for details)^45^ were performed to determine the elastic moduli of neat and doped polymer films. Neat films of P0–P59 with a thickness of 2–8 µm had elastic moduli in the range of *E* = 100 to 300 MPa (Fig. 6b and Table S5), which are typical values for semi-crystalline conjugated polymers above their glass transition temperature *T*_g_ (note that P0 has a *T*_g_ = −5 °C).^40^ Chemical doping with F_4_TCNQ resulted in a significant increase in the elastic modulus to *E* = 0.7 to 1 GPa, similar to polythiophenes with oligoether side chains.^16,46^

**Fig. 7:**
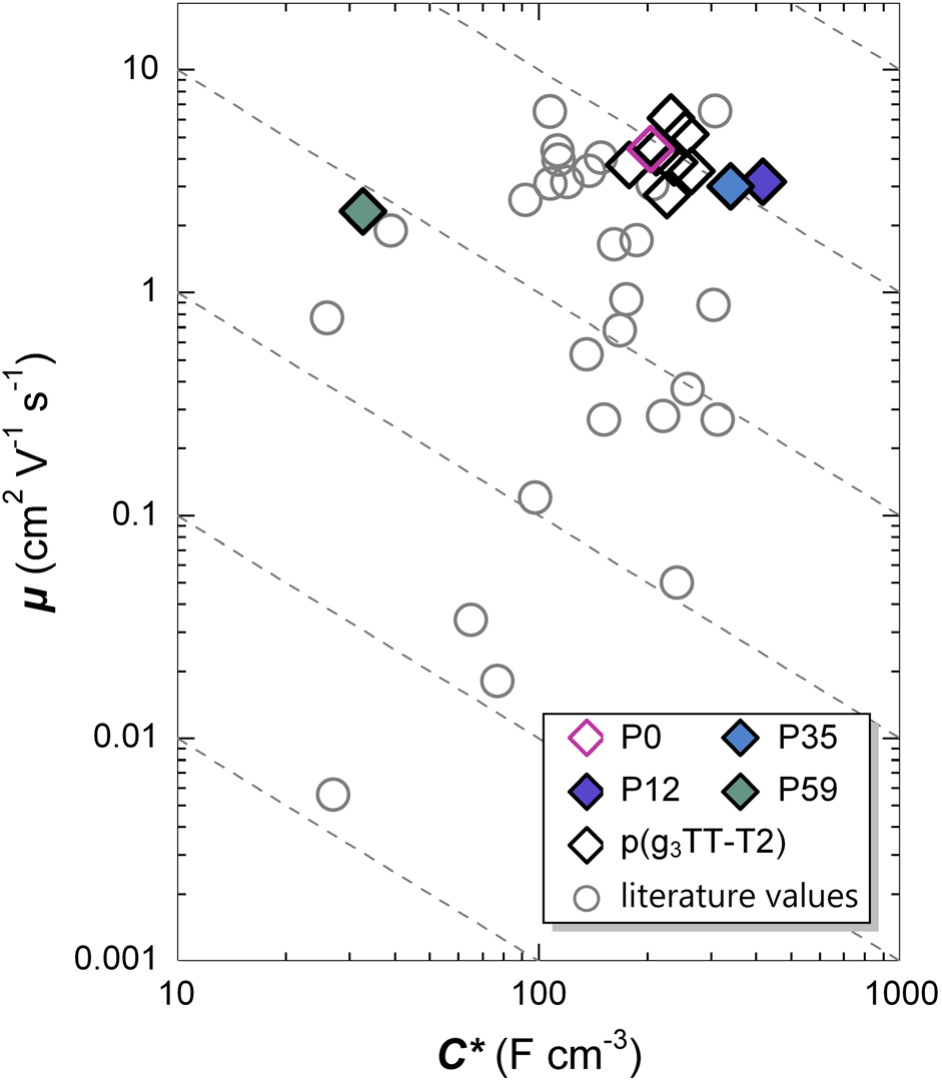
OECT device performance. Charge-carrier mobility *µ vs.* volumetric capacitance C* determined with small-signal analysis at a gate potential of −0.6 V *vs.* Ag/AgCl^47^ of polymers studied in this work (solid diamonds), previously reported values for p(g_3_TT-T2) made by DAP or ADAP (open diamonds)^31,33^ and other literature values (open circles).^33^

OECT devices of P12–P59 were fabricated (see Experimental for details) and transfer curves were recorded in a three-electrode configuration using a 0.1 M NaCl aqueous electrolyte (Fig. 7 and Table 3; see Fig. S16–S19 for device characteristics measured by small signal analysis and Fig. S20 and Table S4 for steady-state transfer curve characterization in the linear regime). The threshold voltage shifted to lower gate potentials *V*_GS_ (= −offset potential *vs.* Ag/AgCl) with increasing alkoxy side-chain fraction, which can be explained with the increase in *E*^CV^_ox_ observed in CV measurements (Table 2). P12 and P35 show a similar charge-carrier mobility of *µ* = 3.2 cm^2^ V^−1^ s^−1^ and 3.0 cm^2^ V^−1^ s^−1^ at *V*_GS_ = −0.6 V, which is comparable to values reported for P0 (Fig. 7 and Table 3)^33^ and in agreement with the *µ* value of their F_4_TCNQ-doped films. In contrast, P59 featured a lower value aligning with the lower *µ* value of F_4_TCNQ-doped films. Strikingly, P12 reach a high volumetric capacitance of C* = (417 ± 14) F cm^−3^ at *V*_GS_ = −0.6 V, which is twice as large as values reported for P0 as well as other high-mobility p-type accumulation mode materials.^11,29,31,33^ As a result, P12 features a high figure of merit [µC*] = (1313 ± 170) F cm^−1^ V^−1^ s^−1^ at *V*_GS_ = −0.6 V, which is comparable to other state-of-the-art materials (Fig. 7). Interestingly, NDI-based n-type polymers with mixed oligoether/alkoxy side chains were found to feature the highest transconductance for a similar content of the alkoxy side-chain bearing comonomer of 10%.^27^

**Table 3:** OECT device performance. Charge-carrier mobility *µ*, volumetric capacitance C* and figure of merit [µC*] recorded with small signal analysis at gate potential *V*_GS_ = −0.6 V (see SI Fig. S20 and Table S4 for steady-state transfer curve characterization in the linear regime)

Polymer	*µ* (cm^2^ V^−1^ s^−1^)	C* (F cm^−3^)	[µC*] (F cm^−1^ V^−1^ s^−1^)
P0^a^	4.4 ± 0.2	204 ± 8	899 ± 47
P12^b^	3.2 ± 0.5	417 ± 14	1313 ± 170
P12^c^	4.1 ± 0.1	299 ± 2	1227 ± 7
P35^b^	3.0 ± 0.3	340 ± 45	1020 ± 167
P59^b^	2.3 ± 0.4	33 ± 3	77 ± 20

a From entry 9 in ref. 33.

b Stored in air for 2 weeks after synthesis.

c Stored in air for 1.5 years; the mean and standard deviation based on a comparison of three or four cycles of two devices are given.

The OECT device performance of P12 was characterized again after storage in air for 1.5 years (Table 3 and Fig. S19). A 6.5% decrease in the figure of merit was observed showing the material is relatively stable in air. A previous report has shown that OECTs based on OMIECs with mixed alkoxy/oligoether side chains can feature increased cycling stability.^26^ We investigated the stability of a P12 device at a scan rate of 10 mV s^−1^ over 100 cycles with the gate potential *V*_GS_ varying between +0.4 and −0.6 V (Fig. S21). A 10% decrease in the drain-source current at *V*_GS_ = −0.6 V was observed after 37 cycles, which indicates that P12 is less stable than p(g_3_TT-T2).^31^ We conclude that for the here studied materials the introduction of alkoxy side chains does not necessarily improve the OECT cycling stability.

## Conclusions

A series of random copolymers bearing both alkoxy and oligoether side chains, p[(g_3_TT-T2)-*ran*-(a_10_TT-T2)], was synthesized through a modified ADAP method that accommodates the solubilities of both oligoether moieties and less polar alkoxy moieties. An extensive analysis of their chemical structures was conducted with high-temperature ^1^H NMR spectroscopy. The ratio of a_10_TT to g_3_TT units (and therefore alkoxy to oligoether side chains) in the random copolymers was found to be governed by the monomer feed ratio with a slight preference for a_10_TT incorporation. End-group analysis revealed that copolymers with an alkoxy side-chain fraction of up to 59% feature a number-average molecular weight above 10 kg mol^−1^, while a higher ratio resulted in a significantly lower degree of polymerization.

Copolymers with an alkoxy side-chain fraction of 12 to 35% featured a high electrical conductivity of up to 400 S cm^−1^ upon chemical doping with F_4_TCNQ. OECT devices comprising copolymer active layers revealed a figure of merit of up to [µC*]_max_ = (1313 ± 170) F cm^−1^ V^−1^ s^−1^ for the copolymer with an alkoxy side-chain fraction of 12%. We conclude that replacing a minor fraction of the oligoether side-chain bearing monomer with an alkoxy functionalized comonomer is an effective approach for improving the electrical and electrochemical performance of OMIECs based on conjugated polymers with oligoether side chains.

## Experimental

### Materials

The synthesis of the a_10_TT monomer and ambient direct arylation polymerization yielding the p[(g_3_TT-T2)-*ran*-(a_10_TT-T2)] random copolymers is described in the SI. Reactants, chemical dopants and solvents were purchased from TCI, Sigma Aldrich, Fisher Scientific and Thermo Scientific and were used without further purification unless stated otherwise (see SI for details).

### Film preparation

Thin films with a thickness of 20–40 nm were spin-coated from solutions of 5–8 g L^−1^ polymer dissolved in argon-purged chloroform onto the desired substrate; the substrate for CV and UPS was a pre-cleaned (by washing with soap and deionized water, sonication in acetone and then in isopropyl alcohol, and blow drying with pressured air or N_2_) ITO-coated glass slide (Ossila, 20 Ω cm^−2^), the substrate for UV-Vis-NIR spectroscopy and electrical characterization was a pre-cleaned (by sonication in acetone and then in isopropanol, and blow drying with pressured air or N_2_) microscope glass slide, and the substrate for GIWAXS was a pre-cleaned (by sonication in acetone and then in isopropanol, and spin-coated at 3000 rpm s^−1^ with toluene) silicon wafer. Films for UPS were spin coated under N_2_ atmosphere. Films for UV-Vis-NIR spectroscopy were annealed at 45 °C for 10 to 15 min. Sequential doping was performed by spin-coating (1500 rpm, 500 rpm s^−1^, 30 s) 90 µL F_4_TCNQ solution (5 mM in acetonitrile : ethyl acetate = 95 : 5) onto the polymer films. The film thickness was measured using a Sensofar S neox optical profilometer in interferometry mode. Thick films with a thickness of 2–8 µm were prepared by drop casting 150 µL polymer solution (20 g L^−1^ in argon-purged chloroform) in a PDMS mold (1 cm diameter) on pre-cleaned (by sonication in acetone and then in isopropanol, and blow drying with N_2_) microscope glass slides at room temperature. Thick films were sequentially doped by placing them in a F_4_TCNQ solution (5 mM in argon-purged acetonitrile) for 3 hours followed by drying in a vacuum oven overnight at 40 °C.

### High-temperature nuclear magnetic resonance (NMR)

High-temperature ^1^H NMR spectra were recorded on an Agilent Technologies 400 MR spectrometer (^1^H: 400 MHz) and Bruker Ascend Evo 400 (^1^H: 400 MHz). The ^1^H spectra were referenced to the residual solvent peak (C_2_D_2_Cl_4_: *δ*(^1^H) = 6.0 ppm). The molecular weight and alkoxy side-chain fraction of copolymers were determined from high-temperature measurements in C_2_D_2_Cl_4_. The obtained spectra were analyzed with MestReNova.

### Size exclusion chromatography (SEC)

Chromatograms were recorded at 308 K with analytical GPC measurements using an Agilent LC1260 Infinity II instrument. The system utilized three SDV columns (8 × 300 nm, 5 µm, PSS) connected in series as the stationary phase. Chloroform (Thermo Scientific, 99+%, for HPLC, stabilized with ethanol) with 0.5 vol% triethylamine (Fisher Scientific, ≥ 99.5%, HPLC grade) was used as the mobile phase, eluting at 1 mL min^−1^. Polymer samples were prepared by dissolving them in the eluent to a concentration of 1 g L^−1^ followed by filtration over 0.45 µm glass fiber membranes prior to injection. Molecular weight values are estimated against narrow polystyrene standards (474–2 520 000 g mol^−1^).

### Cyclic voltammetry (CV)

Cyclic voltammograms were recorded at a scan rate of 10 mV s^−1^ in a nitrogen-purged 0.1 M NaCl aqueous electrolyte using a three-electrode configuration (Ag/AgCl reference electrode (3 M KCl) and Pt wire as the counter electrode) and a SP-300 electrochemical workstation from BioLogic.

### Ultraviolet photoelectron spectroscopy (UPS)

Ultraviolet photoelectron spectroscopy was performed on a home-built spectrometer using monochromatic He I radiation (*hν* = 21.22 eV). The work function was determined from the secondary-electron cutoff, and the ionization potential from the leading edge of the occupied density of states. The overall energy uncertainty is ± 0.05 eV.

### UV-Vis-NIR absorption

A PerkinElmer Lambda 1050 spectrophotometer was used to record UV-Vis-NIR spectra of both neat and doped thin films with a thickness of 25–50 nm.

### Electrical characterization

The electrical resistance of thin films was measured using a four-point probe setup from Jandel Engineering (cylindrical probe head, RM3000) using collinear tungsten carbide electrodes at regular spacing of 1 mm. The electrical conductivity was derived according to *σ*_thin_ = ln 2/(π*Rd*) where *R* is the measured resistance and *d* is the film thickness. The electrical conductivity of micrometre-thick films was determined using the van der Pauw method for isotropic square samples.^48^ Measurements were performed using a Tektronix Keithley 2400 Source Meter.

### Grazing incidence wide-angle X-ray scattering

GIWAXS patterns were recorded at the beamline NCD-SWEET of the Alba synchrotron light source facility using an X-ray wavelength of 1 Å and a sample-detector distance of 201.17 cm.

### Mechanical characterization

Nanoindentation was carried out at room temperature with a Hysitron TI Premier instrument from Bruker equipped with a Berkovich tip made of diamond with a half angle of 65.27°, calibrated with a reference quartz substrate. The maximum allowed drift during all experiments was 0.02 nm s^−1^, resulting in a less than 0.5% error in indentation depth. Creep experiments were performed at a load of 60–2000 µN, loading time of 500–600 s, and a loading rate of 5–50 µN s^−1^.^45^

### Organic electrochemical transistor (OECT) fabrication and device characterizations

Source and drain metal electrodes were defined *via* a conventional lift-off process using a Karl Suss MA6 contact aligner and a Kurt J Lesker PVD e-beam evaporator on cleaned Marienfeld soda lime glass slides, resulting in channels with a length *L* = 20 µm. The two parylene films were sequentially deposited with a thickness of 300 nm and 1 µm with an anti-adhesive soap layer between them. Two parylene films were patterned *via* a conventional dry-etching process using a Karl Suss MA6 contact aligner and reactive ion etcher (O2, 300 W), resulting in a channel width *w* = 100 µm. Then, the active layer was spin-coated from a solution of 8 g L^−1^ polymer dissolved in chloroform onto the patterned substrate, followed by peeling away of the second parylene film to pattern the active layer and soft baking for 10 minutes at 45 °C.

Small signal analysis of OECTs was conducted with a SP-300 two-channel electrochemical workstation from BioLogic, as described previously.^47^ A mixed gate potential with a pseudo steady-state triangular potential (scan rate = 10 mV s^−1^) and a sinusoidal AC potential (amplitude = 10 mV, frequency = 10 Hz) was applied using a three-electrode configuration with a Pt gate and an Ag/AgCl reference electrode.

Steady-state transfer curve characterization was conducted with two Matlab-controlled Keithley 2400 source-measure units. For the gate electrode the built-in ‘four-wire mode’ function of the source-measure unit was used. The Ag/AgCl reference electrode and Pt counter electrode, which were immersed in the electrolyte, were electrically connected to the HP and HC ports of the source-measure unit, respectively, and the LP and LC ports were connected to the source electrode (HP: high potential, HC: high current, LP, low potential, LC: low current). For the drain-source potential, drain and source electrodes were connected to the high and low ports of the other source-measure unit using a conventional ‘two-wire mode’. The applied potential values for steady-state transfer curve characterization and small signal analysis are in the linear regime where a drain-source voltage *V*_DS_ = +0.01 V was applied, unless otherwise specified.

## Author contributions

D. Z. conceived the study and wrote the manuscript together with C. M., and carried out the synthesis and chemical characterizations. J. P. T. carried out CV measurements, fabricated and characterized OECT devices and carried out small signal analysis. J. P. T. and T. C. H. carried out EIS, steady-state transfer curve characterizations and stability tests. J. K. aided synthesis and supervised chemical characterizations. M. J. carried out nanoindentation measurements and thick film conductivity measurements and processed the data. M. C. carried out the chemical doping of thin films, UV-Vis measurements and thin film conductivity measurements. J. A. and A. P. V. carried out GIWAXS measurements and processed data together with Y. K.; J. M. supervised the GIWAXS analysis. Z. L. carried out UPS measurements; M. F. supervised the UPS analysis. M. M. W. carried out SEC measurements; A. G. supervised SEC measurements and co-supervised the project. All authors reviewed and edited the initial draft. C. M. supervised the overall study.

## Conflicts of interest

The authors declare no conflict of interest.

## Supplementary Material

SC-OLF-D6SC01881E-s001

## Data Availability

The data used in this study are available in the zenodo database at https://doi.org/10.5281/zenodo.19701365. Supplementary information (SI) is available. See DOI: https://doi.org/10.1039/d6sc01881e.
