# Supplementary material for: Ambient direct arylation synthesis of thienothiophene based copolymers with mixed alkoxy and oligoether side chains
Source: Chem Sci. 2026 May 26;17(26):13030–41. doi: 10.1039/d6sc01881e (PMC13202795; doi:10.1039/d6sc01881e)
Supplement: SC-017-D6SC01881E-s001 [file SC-017-D6SC01881E-s001.pdf]

Supplementary Information (SI) for Chemical Science.
This journal is © The Royal Society of Chemistry 2026

Supplementary Information

Ambient Direct Arylation Synthesis of Thienothiophene Based Copolymers with Mixed Alkoxy and Oligoether Side Chains

Di Zhu[a], Judith Pons i Tarrés[a], Joost Kimpel[a], Meghna Jha[a], Mariavittoria Craighero[a], Jesika Asatryan[b], Alberto Peinador Veiga[b], Zesheng Liu[c], Tania Cecilia Hidalgo[a], Megan Westwood[a], Mats Fahlman[c], Jaime Martín[b], Alexander Giovannitti[a], Christian Müller[a]*

[a]Department of Chemistry and Chemical Engineering, Chalmers University of Technology, 41296 Gothenburg, Sweden

e-mail: christian.muller@chalmers.se

[b]Universidade da Coruña, Campus Industrial de Ferrol, CITENI, Esteiro, Ferrol, Spain

[c]Laboratory of Organic Electronics, Department of Science and Technology, Linköping University, Norrköping, Sweden

Contents

S1. Experimental details 3

S1.1. Chemicals 3

S1.2. Analytical techniques and device fabrication 4

S1.3. Monomer synthesis 6

S1.4. Polymer synthesis 11

S1.5. Molecular weight derivation by NMR 16

S1.6. Molecular weight estimation by SEC 17

S2. Synthetic complexity index of polymerization (SCI_{poly}) 18

S3. Polymer characterization 20

S3.1. Ultraviolet photoelectron spectroscopy (UPS) 21

S3.2. Electrochemical characterization 22

S3.3. Grazing incidence wide-angle X-ray scattering (GIWAXS) 28

S3.4. Electrical and mechanical properties 29

S4. References 30

S1. Experimental details

S1.1. Chemicals

Analytical grade *n*-hexanes (Fisher Scientific, ≥ 99.5 %), ethyl acetate (Fisher Scientific, ≥ 99.5 %), methanol (MeOH, Fisher Scientific, ≥ 99.9 %), isopropanol (Fisher Scientific, ≥ 99.9 %), chloroform (Fisher Scientific, ≥ 99.8 %), acetonitrile (Fisher Scientific, ≥ 99.8 %), chlorobenzene (Sigma, ≥ 99.5 %), *N*-butyl-2-pyrrolidone (NBP, Sigma Aldrich, ≥ 99.5 %), 3,6-dibromothieno[3,2-*b*]thiophene (Combi-block, 98 %), 5,5'-dibromo-2,2'-bithiophene (Br-T2-Br, TCI, > 98.0 %), triethylene glycol monomethyl ether (Sigma Aldrich, 95 %), decan-1-ol (Sigma Aldrich, 98%), sodium hydride (Sigma Aldrich, 60 %, dispersion in mineral oil) and copper(I) iodide (Sigma Aldrich, ≥ 99.5%) palladium acetate ($[Pd(OAc)_2]_3$, Sigma Aldrich, 98 %) and potassium carbonate (Sigma Aldrich, ≥ 99.0 %) and F_4TCNQ (TCI, > 98%) were used as received. Anhydrous 2-methyltetrahydrofuran (2-MeTHF, Thermo Scientific, ≥ 99.0 %) and anhydrous dimethylacetamide (DMAc, Thermo Scientific, 99.8 %) were taken from their vessels under a constant stream of nitrogen.

S1.2. Analytical techniques and device fabrication

Room temperature nuclear magnetic resonance (NMR). Room temperature spectra were recorded on an Oxford 800 magnet, Bruker Avance III HD spectrometer (^{1}H: 800 MHz, ^{13}C: 201 MHz). The ^{1}H and ^{13}C NMR spectra were referenced to the residual solvent peak ($CDCl_3$: $\delta(^{1}H) = 7.26$ ppm, $\delta(^{13}C) = 77.16$ ppm).

Single crystal X-ray crystallography. The monomer crystal structures were obtained by mounting suitable crystals on a nylon loop on an XtaLAB Rigaku Synergy R, HyPix diffractometer using CuKα radiation ($\lambda = 1.54184$ Å). The crystals were kept at a steady temperature T = 116.0(2) K during data collection. Their structures were solved with the ShelXT21 structure solution program using the Intrinsic Phasing solution method and by using Olex2 as the graphical interface.[1, 2]

Organic electrochemical transistor (OECT) fabrication and small signal analysis. The gate and drain channels were grounded via the 'CE to ground' mode in the EC-Lab software, which allows voltage to be measured based on the source electrode. The gate was operated with a three-electrode setup, while the drain used a two-electrode setup. The Ag/AgCl and Pt electrodes were connected to the S1 and P1 ports for applying gate potential, with the source electrode connected to the S2, S3, and GND ports (henceforth, S1: high potential, P1: high current, S2 and S3: low potential, GND: ground). For the drain potential, the drain electrode was connected to the P1 and S1 ports, and the source electrode to the S2, S3, and GND ports. The gate potential was controlled using the 'AC-Voltammetry' function (a triangular potential with a small sinusoidal waveform), and the drain potential using the 'Constant-Voltage' function (constant voltage bias). The 'synchronize' function in the software was used to coordinate measurements on both channels. Electrical and electrochemical parameters were calculated with MATLAB software.[3]

The resistance of the metal line patterns R_{line} were comparable with the channel resistance R_{ch}, and hence the parasitic resistance values were corrected with the measured R_{line} value.[3] In case of OECT devices that have a very high on-current value, the channel resistance does not equal the input resistance R_{in} (i.e., the total resistance of a device including the channel resistance R_{ch} as well as the line resistances of the source and drain electrodes, $R_{line,S}$ and $R_{line,D}$). Thus, the voltage across the channel V_{ch} is smaller than the applied voltage V_{DS}. Hence, all parameters were recalculated with the corrected drain current by using the correct voltage experienced by the channel, assuming no parasitic resistance between the two drain and source contact pads according to ref. [3].

S1.3. Monomer synthesis

3,6-bis(decyloxy)thieno[3,2-b]thiophene (a_{10}TT)

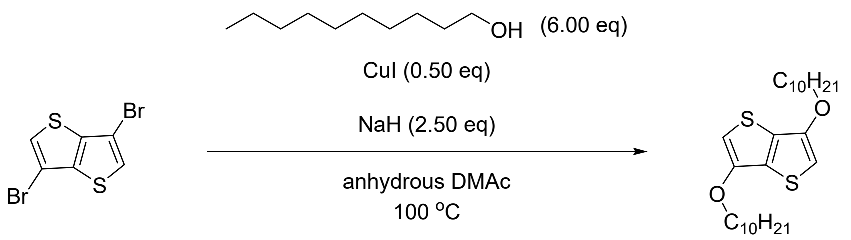

Fig. S1. Reaction scheme of 3,6-bis(decyloxy)thieno[3,2-*b*]thiophene (a_{10}TT).

Decan-1-ol was dried over molecular sieves and purged with argon before the reaction. Anhydrous DMAc (6.70 mL) was charged into a dried two-neck round bottom flask equipped with a condenser on one neck and a rubber septum on the other, under nitrogen atmosphere. The system was cooled down to ~ 0 ºC with an ice bath. While stirring, 60% mineral suspension of sodium hydride (1.01 g, 25.17 mmol, 2.50 eq) was added, and the dispersion is brought back to the nitrogen atmosphere. Dried decan-1-ol (11.52 mL, 60.40 mmol, 6.00 eq) was added dropwise to the system. Once the decan-1-ol was depleted, the mixture was slowly heated to 35 ºC and left to stir until no more bubble formation was observed. To avoid the formation of sodium decan-1-oxide, which can lead to solidification of the system, the temperature was slightly increased (< 50 ºC) and/or 2 – 3 mL anhydrous DMAc was added to dilute the system, where necessary. Solids of 3,6-dibromothieno[3,2-*b*]thiophene (3.00 g, 10.07 mmol, 1.00 equiv.) and copper iodide (958.61 mg, 5.03 mmol, 0.50 equiv.) were mixed and added to the solution simultaneously. Then, the reaction mixture was slowly heated up to 100 ºC (5-10 ºC every 10 min). The reaction was monitored using thin layer chromatography with hexane as the eluent (R_f = 0.21). After 24 hours, the reaction was cooled to room temperature and filtered to remove residual solids. The residual solids were washed with 2 × 50 mL of ethyl acetate. The combined filtrates were washed with 3 × 100 mL saturated NH_4Cl solution. The combined aqueous phase was extracted with 3 × 150 mL ethyl acetate. The combined organic phase was

dried over anhydrous $MgSO_4$. The mixture was concentrated *in vacuo* to yield a dark brown crude oil, which slowly crystalized. The crude was dissolved in a minimal amount of ethyl acetate and passed through a silica gel column (eluent: hexane → 5 vol% ethyl acetate in hexane). The obtained product was recrystallized in hexane at -20 ºC to yield off-white crystals (2.06 g, 4.55 mmol, 45%). ^{1}H NMR (800 MHz, $CDCl_3$) δ 6.23 (s, 2H), 4.05 (t, J = 6.6 Hz, 4H), 1.85 – 1.77 (m, 4H), 1.50 – 1.42 (m, 8H), 1.39 – 1.24 (m, 8H), 0.88 (t, J = 7.0 Hz, 6H). ^{13}C NMR (201 MHz, $CDCl_3$) δ 150.2, 128.6, 97.8, 70.6, 32.0, 29.6, 29.4, 29.4, 29.2, 26.0, 22.7, 14.2.

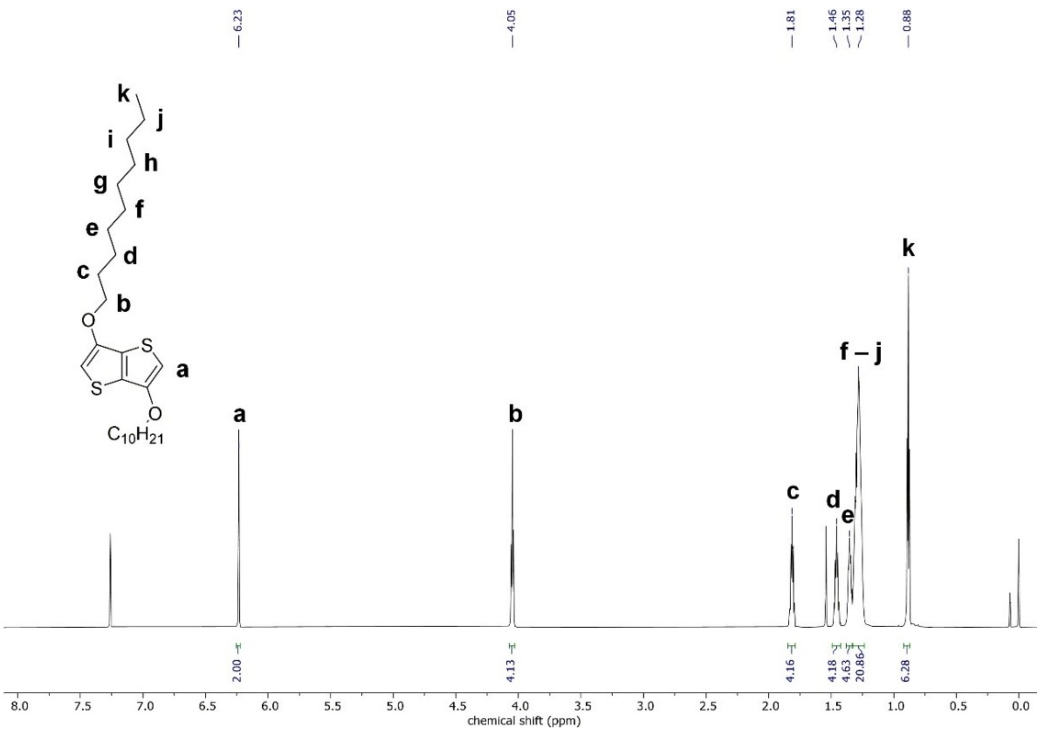

Fig. S2. ^{1}H NMR spectrum ($CDCl_3$, 800 MHz, 298K) of 3,6-bis(decyloxy)thieno[3,2-*b*]thiophene.

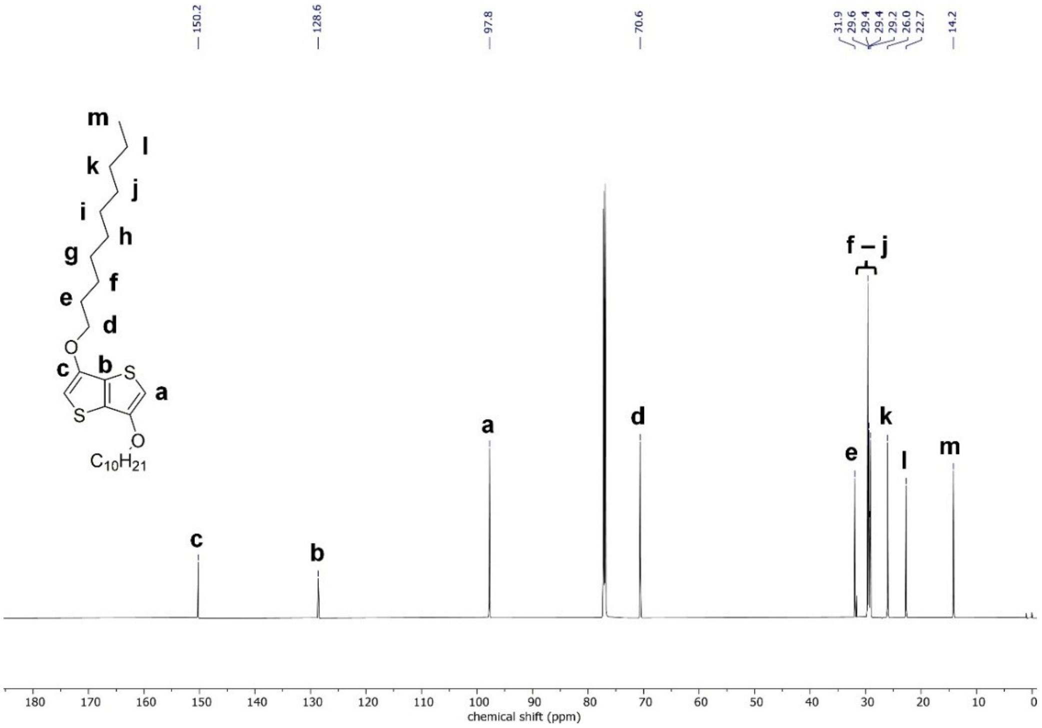

Fig. S3. ^{13}C NMR spectrum ($CDCl_3$, 201 MHz, 298K) of 3,6-bis(decyloxy)thieno[3,2-*b*]thiophene.

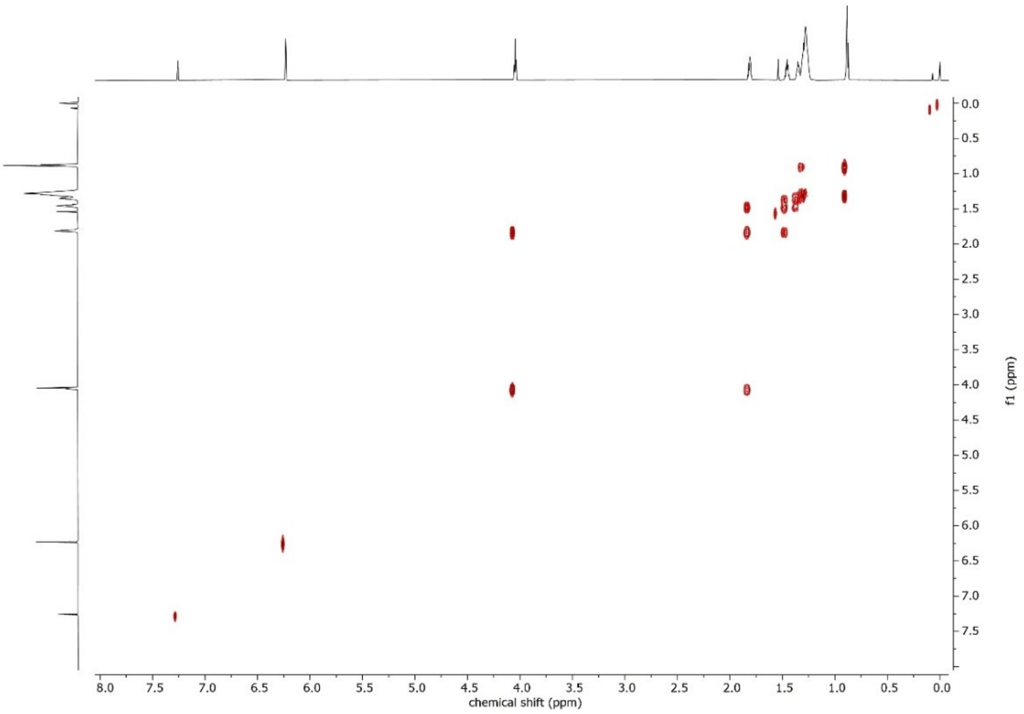

Fig. S4. ^{1}H COSY NMR spectrum ($CDCl_3$, 800 MHz, 298K) of 3,6-bis(decyloxy)thieno[3,2-*b*]thiophene.

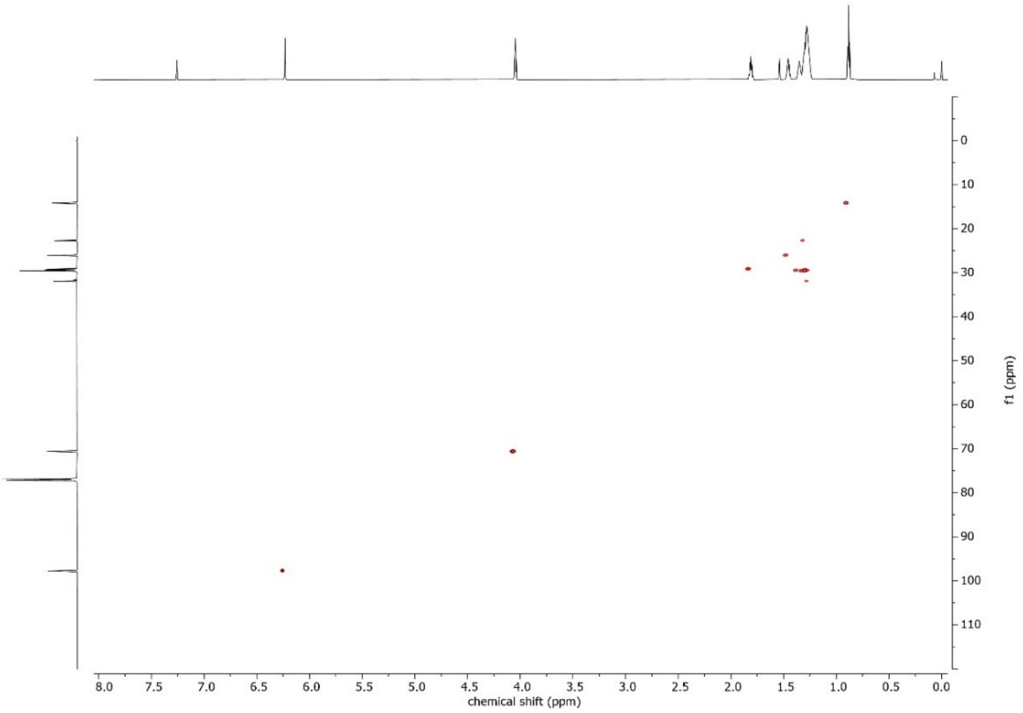

Fig. S5. HSQC NMR spectrum ($CDCl_3$, 800/201 MHz, 298K) of 3,6-bis(decyloxy)thieno[3,2-*b*]thiophene.

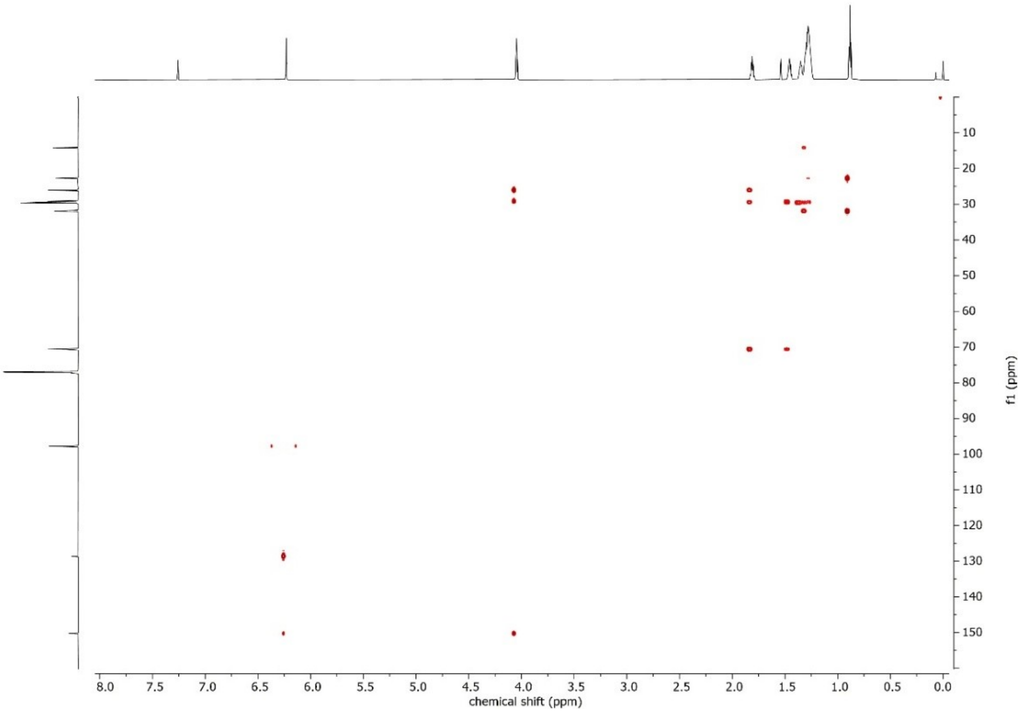

Fig. S6. HMBC NMR spectrum ($CDCl_3$, 800/201 MHz, 298K) of 3,6-bis(decyloxy)thieno[3,2-*b*]thiophene.

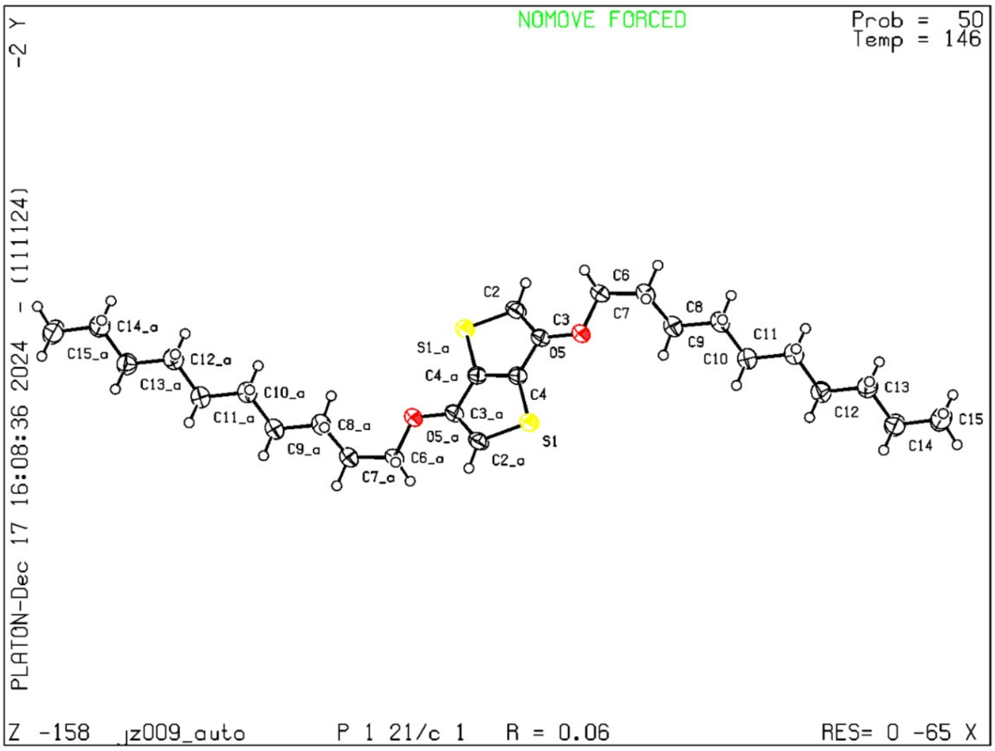

Fig. S7. Single crystal X-ray ellipsoid plot of 3,6-bis(decyloxy)thieno[3,2-*b*]thiophene (CCDC = 2503701). Further crystallographic data can be obtained from CCDC or ref. [4].

S1.4. Polymer synthesis

General procedure of polymer synthesis

a_{10}TT (R eq., **Table S1**), g_3TT ($1-R$ eq., **Table S1**), Br-T2-Br (1 eq) and K_2CO_3 (3 eq) were charged into a 10 mL high pressure reaction vial. The solid mixture underwent three N_2/vacuum cycles and was then kept under vacuum for 30 minutes. A stock solution of 2.5 mM $Pd[(OAc)_2]_3$ in argon-purged NBP was freshly made under argon atmosphere by vigorously stirring at 45 ºC for 30 minutes. After the reaction vial was filled with argon, the required amount of stock solution (0.05 eq of $Pd[(OAc)_2]_3$) was added to the solids. The reaction mixture was heated to 45 ºC and vigorously stirred for 14 hours. Reaction progress was monitored by precipitation of aliquots in isopropanol, ethyl acetate and chloroform. Once precipitates formed in isopropanol and ethyl acetate, the reaction was considered complete. The mixture was precipitated into methanol. Solids were collected by filtration using filter paper. Soxhlet extraction was performed with isopropanol, ethyl acetate to remove the impurities and lower molecular weight fractions and chloroform to extract the solution processable polymer product. Chloroform was removed from the product *in vacuo*. The collected product was dried in a vacuum oven overnight at 50 ºC.

Table S1. Stoichiometry table of synthesis of p[(g_3TT-T2)-*ran*-(a_{10}TT-T2)] and p(a_{10}TT-T2).

	a_{10}TT			g_3TT			Br-T2-Br			K_2CO_3		$Pd(OAc)_2$ stock solution	Product	
Polymers	R (eq.)	Mass (mg)	Moles (μmol)	$1 - R$ (eq.)	Mass (mg)	Moles (μmol)	Mass (mg)	Moles (μmol)	Concentration (mM)	Mass (mg)	Moles (μmol)	Volume (mL)	Mass (mg)	Yield (%)
P12	0.1	7.0	15.4	0.9	64.5	138.9	50	154.3	50	64.0	462.9	3.1	69	72
P35	0.3	21.0	46.3	0.7	50.2	108.0	50	154.3	50	64.0	462.9	3.1	40	42
P59	0.5	27.9	61.7	0.5	28.7	61.7	40	123.4	50	51.2	370.3	2.5	22	29
P79	0.7	39.1	86.4	0.3	17.2	37.0	40	123.4	50	51.2	370.3	2.5	36	47
P95	0.9	50.3	111.1	0.1	5.7	12.3	40	123.4	50	51.2	370.3	2.5	31	41
P100	1.0	97.8	216.0	0	0	0	70	216.0	50	89.6	648.1	4.3	52	39

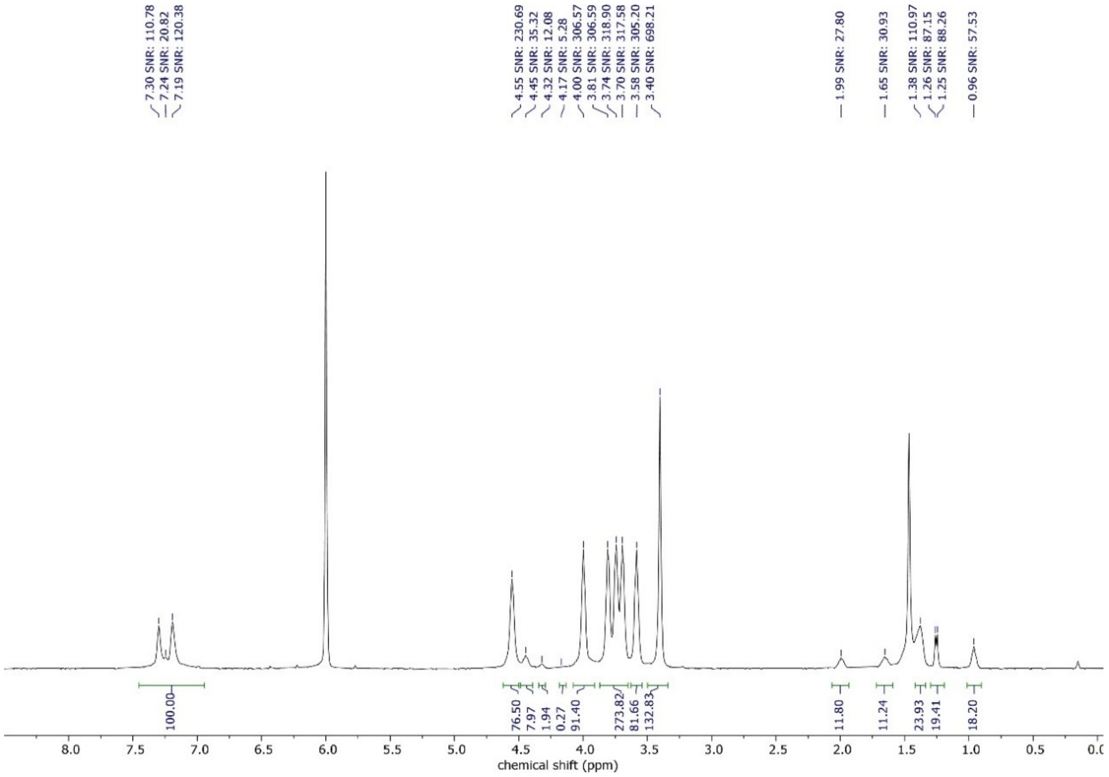

Fig. S8. High-temperature ^{1}H NMR spectrum ($C_2D_2Cl_4$, 400 MHz, 393K) of **P12**.

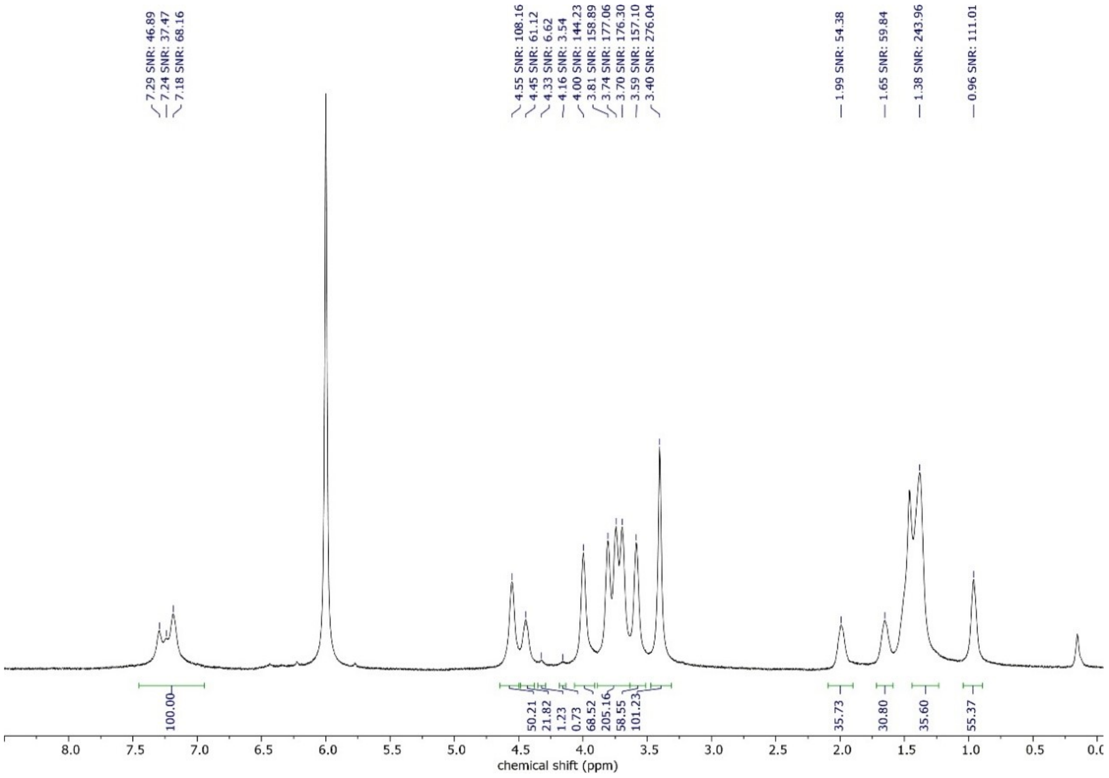

Fig. S9. High-temperature ^{1}H NMR spectrum ($C_2D_2Cl_4$, 400 MHz, 393K) of **P35**.

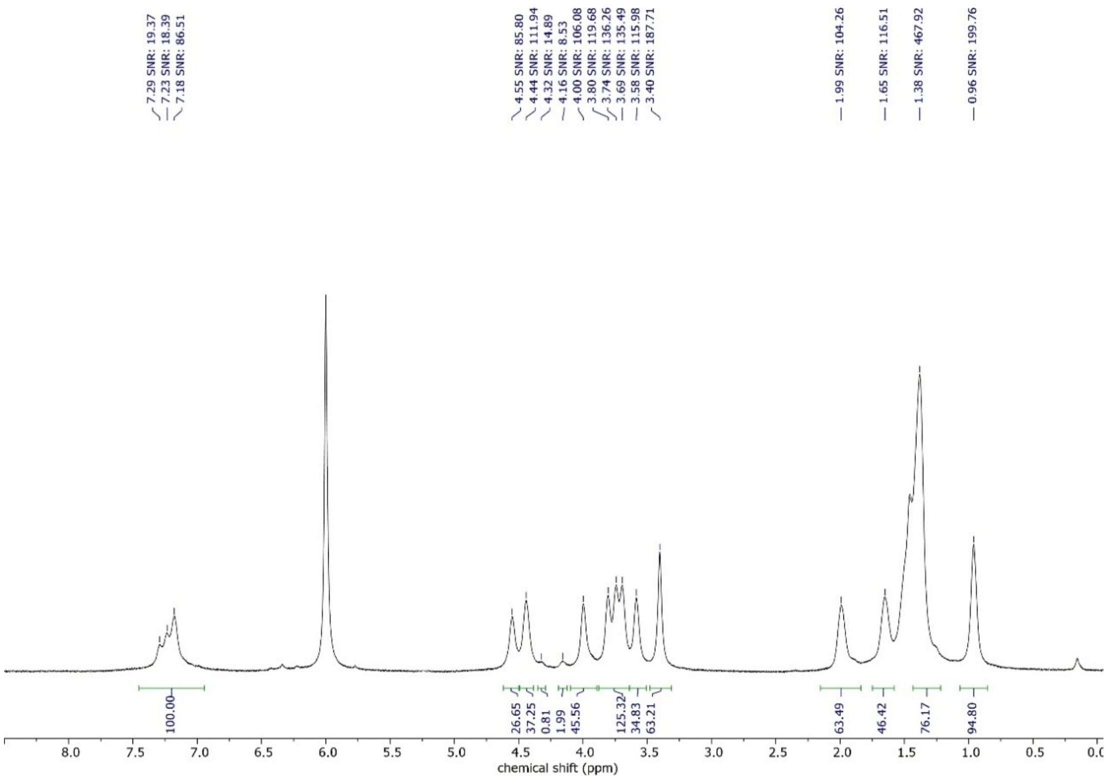

Fig. S10. High-temperature ^{1}H NMR spectrum ($C_2D_2Cl_4$, 400 MHz, 393K) of **P59**.

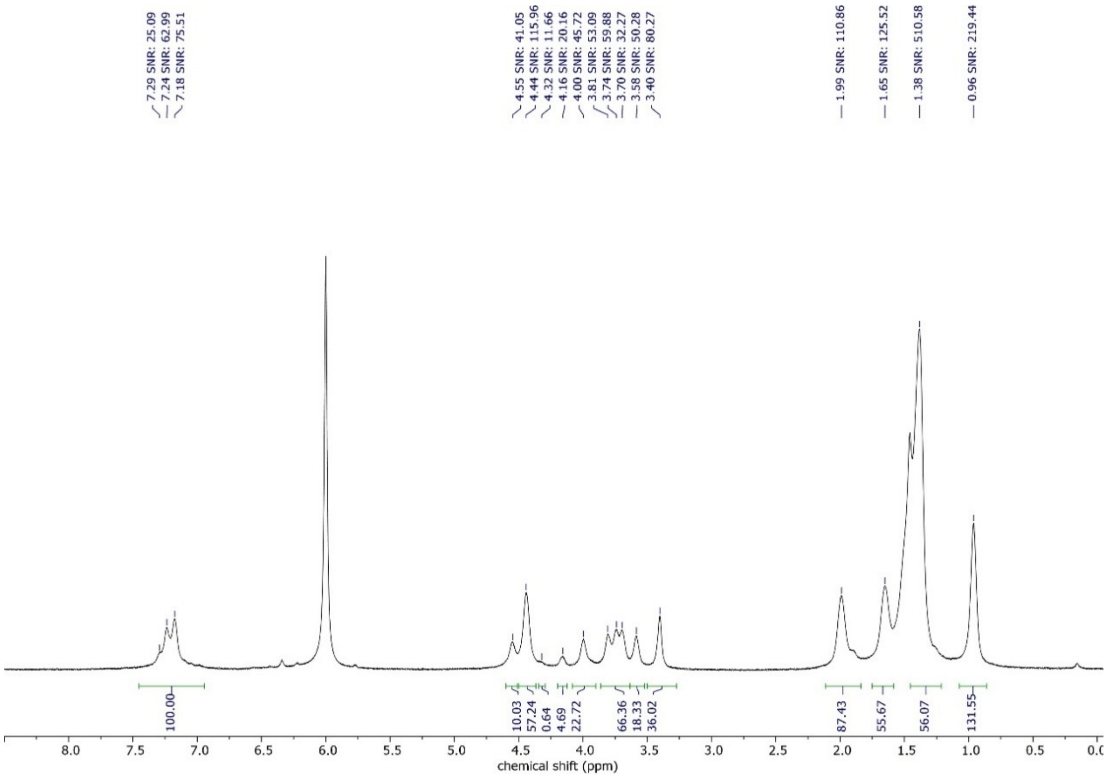

Fig. S11. High-temperature ^{1}H NMR spectrum ($C_2D_2Cl_4$, 400 MHz, 393K) of **P79**.

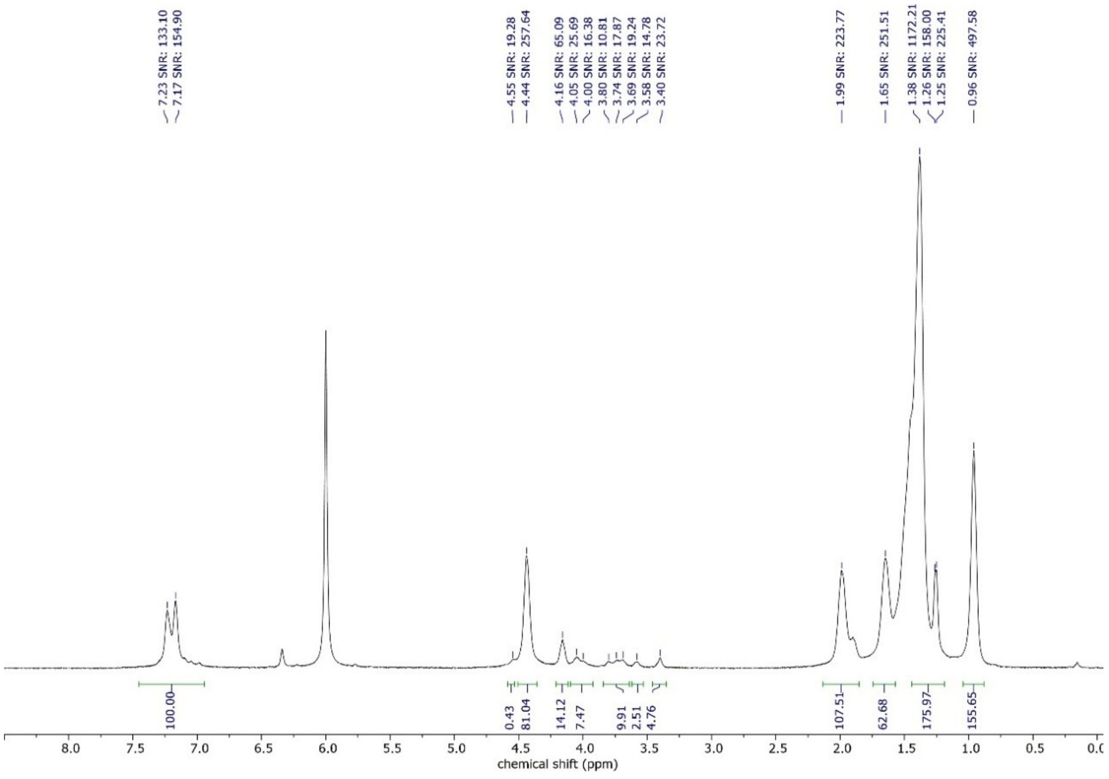

Fig. S12. High-temperature ^{1}H NMR spectrum ($C_2D_2Cl_4$, 400 MHz, 393K) of **P97**.

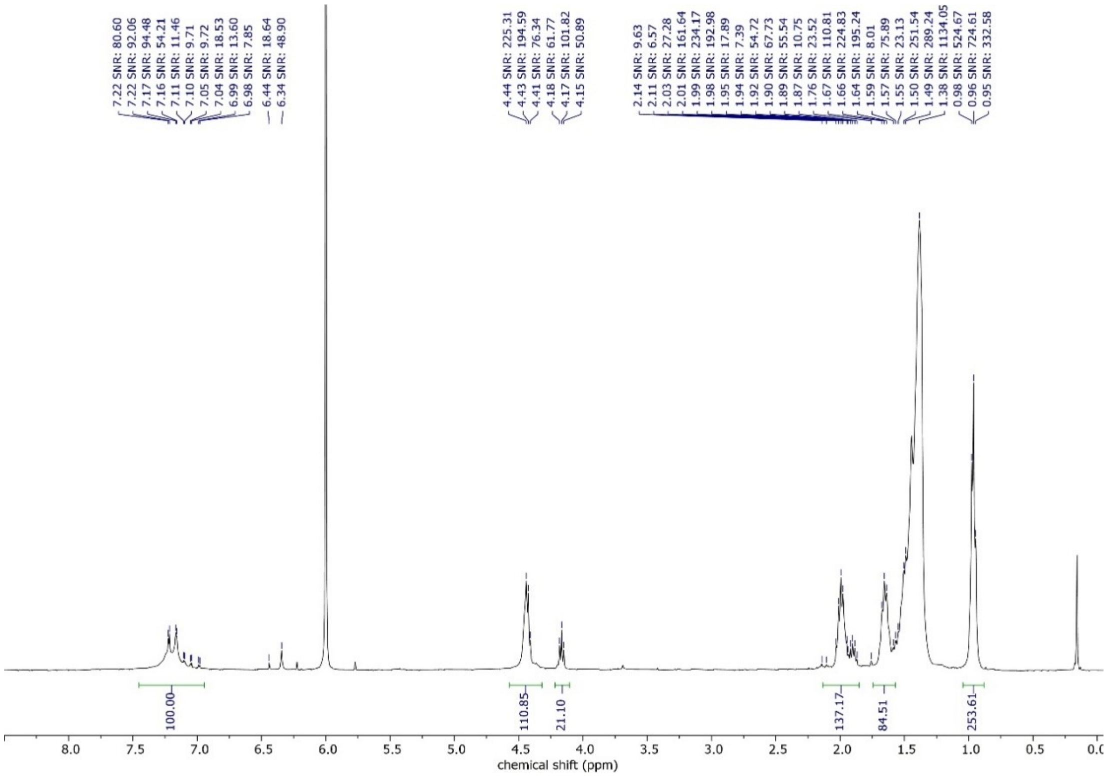

Fig. S13. High-temperature ^{1}H NMR spectrum ($C_2D_2Cl_4$, 400 MHz, 393K) of **P100**.

S1.5. Molecular weight derivation by NMR

The degree of polymerization $X_n^{NMR,j}$ was obtained by calculating the ratio of the total number of TT units to the number of terminal TT units with oligoether or alkoxy side chains according to:

$$X_n^{NMR,j} = \frac{1}{2} \cdot \frac{\int I_{chain}^{j} d\delta + \int I_{end}^{j} d\delta}{\int I_{end}^{j} d\delta} \quad \text{(S1)}$$

where $j = g_3$ or a_{10} denotes signals associated with the α-CH_2 protons of oligoether or alkoxy side chains, respectively, I_{chain}^{j} is the intensity of the signal associated with α-CH_2 from the side chains of main-chain TT units (region I or II in **Fig. 1b**) and I_{end}^{j} is the intensity of the signal associated with α-CH_2 from the side chains of terminal TT units (region III or IV for g_3 or a_{10}, respectively, in **Fig. 1b**). Note that in case of **P97** the α-CH_2 signal from the terminal oligoether chain was overshadowed by the α-CH_2 signal from the main-chain alkoxy chain and thus we were unable to calculate X_n^{NMR,g_3} (**Fig. S12**).

The copolymer-dependent average molecular weight of a TT-T2 repeat unit was determined according to

$$M_{r.u.} = M_{a_{10}TT} \cdot r_{CH_3} + M_{g_3TT} \cdot \left(1 - r_{CH_3}\right) + M_{T2} \quad \text{(S2)}$$

where the two TT units g_3TT and a_{10}TT have a similar molecular weight of M_{g_3TT} = 464.6 g mol^{-1} and $M_{a_{10}TT}$ = 452.8 g mol^{-1}, respectively, r_{CH_3} is the fraction of TT units with alkoxy side chains and M_{T2} is the weight of a bithiophene unit (**Fig. 3b**, **Table 1**).

The number-average molecular weight $M_n^{NMR,j}$ was obtained according to:

$$M_n^{NMR,j} = M_{r.u.} \cdot X_n^{NMR,j} + 2 \cdot M_H \quad \text{(S3)}$$

S1.6. Molecular weight estimation by SEC

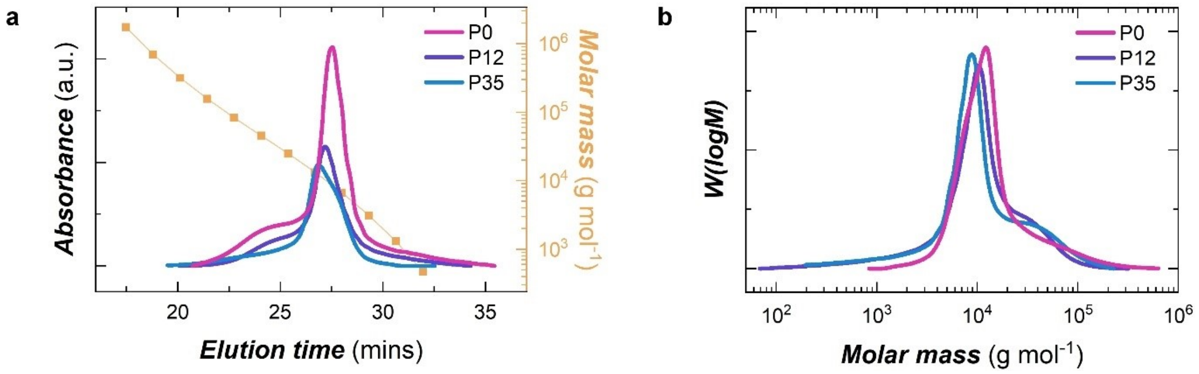

Fig. S14. Size exclusion chromatography (SEC). (a) Elugram with calibration curve (orange) and (b) molar mass distribution of **P0** – **P35**; chromatograms were recorded at 308 K in $CHCl_3$ with 0.5 vol % Et_3N.

Table S2. Molecular weight estimations from SEC. Number-averaged molecular weight M_n^{SEC}, weight-averaged molecular weight M_w^{SEC} and dispersity ***Đ*** of **P0** – **P35** estimated by SEC.

Polymer	M_n^{SEC} (kg mol^{-1})	M_w^{SEC} (kg mol^{-1})	***Đ*** (-)
P0	10	22	2.2
P12	5	16	3.4
P35	5	17	3.4

S2. Synthetic complexity index of polymerization ($\mathrm{SCI_{poly}}$)

The synthetic complexity analysis only considering the polymerization step, i.e., $\mathrm{SCI_{poly}}$, was described in ref. [5]. The $\mathrm{SCI_{poly}}$ calculation is summarized by:

$$\mathrm{SCI_{poly}} = 35 \cdot \frac{NSS}{NSS_{max}} + 25 \cdot \frac{\log RY}{\log RY_{max}} + 15 \cdot \frac{NO}{NO_{max}} + 15 \cdot \frac{NC}{NC_{max}} + 10 \cdot \frac{NH}{NH_{max}} \tag{S4}$$

where NSS is the number of synthetic steps, RY is the reciprocal yield of the total yield, NO is the number of operations, NC is the number of columns, NH is the number of hazard codes and the coefficient of each term is assigned empirically according to overall cost and safety.

Table S3. Input parameters for SCI_{poly} calculations of synthesized polymers and polymers from literature.

	Polymer	SCI_{poly}	Number of Synthetic Steps	Yield	Reciprocal Yield (100/Yield)	Operations: Quenching	Operations: Precipitation	Operations: Extraction	Operations: Soxhlet	Sum of Operations	Number of Columns	Hazards: Carcinogenic / mutagenic (H340, H341, H350, H351)	Hazards: Toxic for reproduction (H360, H361)	Hazards: Highly toxic (H300, H304, H310, H330, H370, H372)	Hazards: Environmentally toxic (H400, H410, H411)	Hazards: Highly reactive with water (H260, H261)	Hazards: Highly flammable (H220, H222, H224, H250)	Hazards: Highly corrosive (H290, H314, H318)	Hazards: Explosive (H200, H201, H202, H203, H204, H205, H240, H241, H271)	Sum of Hazard Codes
Synthesized in this work	P12	50	1	72	1.4	0	1	0	3	4	0	1	2	2	2	0	0	0	0	7
	P35	58	1	42	2.4	0	1	0	3	4	0	1	2	2	2	0	0	0	0	7
	P59	63	1	29	3.4	0	1	0	3	4	0	1	2	2	2	0	0	0	0	7
	P79	56	1	47	2.1	0	1	0	3	4	0	1	2	2	2	0	0	0	0	7
	P97	58	1	41	2.4	0	1	0	3	4	0	1	2	2	2	0	0	0	0	7
	P100	59	1	39	2.6	0	1	0	3	4	0	1	2	2	2	0	0	0	0	7
p(g_3TT-T2) by ADAP	P0	44	1	97	2.1	0	1	0	3	4	0	1	2	2	2	0	0	0	0	7
	p(g_3TT-T2)	46	1	87	2.1	0	1	0	3	4	0	1	2	2	2	0	0	0	0	7
	p(g_3TT-T2)	45	1	92	2.1	0	1	0	3	4	0	1	2	2	2	0	0	0	0	7
	p(g_3TT-T2)	47	1	81	2.1	0	1	0	3	4	0	1	2	2	2	0	0	0	0	7
	p(g_3TT-T2)	50	1	65	2.1	0	1	0	3	4	0	1	2	2	2	0	0	0	0	7
	p(g_3TT-T2)	53	1	54	2.1	0	1	0	3	4	0	1	2	2	2	0	0	0	0	7
	p(g_3TT-T2)	55	1	45	2.1	0	1	0	3	4	0	1	2	2	2	0	0	0	0	7
	p(g_3TT-T2)	48	1	76	2.1	0	1	0	3	4	0	1	2	2	2	0	0	0	0	7
	p(g_3TT-T2) (by DAP)	72	1	33	3.0	1	2	1	4	4	0	3	4	4	4	0	0	0	0	15
TT-T2 polymers by Stille	p(g_3T2-TT)	71	1	47	2.1	1	3	1	5	10	0	2	3	8	3	0	1	0	0	17
	p(g_4T2-TT)	96	1	18	5.6	0	2	0	6	8	1	3	3	8	2	0	0	0	0	16
	p(g_4T2-TT) (no column)	60	1	77	1.3	0	2	0	6	8	0	3	3	8	2	0	0	0	0	16
	pgBTTT	76	1	24	4.2	0	2	0	5	7	0	3	5	7	3	0	0	0	0	18
	pBTTT	56	1	87	1.1	0	2	0	5	7	0	1	3	6	5	0	0	0	0	15
pBTTT-like copolymer series	g-100%	60	1	67	1.5	0	2	0	5	7	0	3	4	6	2	0	0	0	0	15
	g-90%	75	1	25	4.0	0	2	0	5	7	0	3	4	6	2	0	0	0	0	15
	g-75%	75	1	25	4.0	0	2	0	5	7	0	3	4	6	2	0	0	0	0	15
	g-50%	62	1	58	1.7	0	2	0	5	7	0	3	4	6	2	0	0	0	0	15
	g-0%	61	1	65	1.5	0	2	0	5	7	0	3	4	6	2	0	0	0	0	15
NDI-T2 copolymer series	P-0	58	1	77	1.3	0	2	0	5	7	0	1	4	5	4	0	0	0	0	14
	P-10	62	1	55	1.8	0	2	0	5	7	0	1	4	5	4	0	0	0	0	14
	P-25	64	1	50	2.0	0	2	0	5	7	0	1	4	5	4	0	0	0	0	14
	P-50	63	1	54	1.9	0	2	0	5	7	0	1	4	5	4	0	0	0	0	14
	P-75	58	1	76	1.3	0	2	0	5	7	0	1	4	5	4	0	0	0	0	14
	P-90	59	1	70	1.4	0	2	0	5	7	0	1	4	5	4	0	0	0	0	14
	P-100	64	1	48	2.1	0	2	0	5	7	0	1	4	5	4	0	0	0	0	14

Polymer		SCI_{poly}	**Number of Synthetic Steps**	Yield	**Reciprocal Yield (100/Yield)**	Operations				**Sum of Operations**	**Number of Columns**	Hazards								**Sum of Hazard Codes**
						Quenching	Precipitation	Extraction	Soxhlet			Carcinogenic / mutagenic (H340, H341, H350, H351)	Toxic for reproduction (H360, H361)	Highly toxic (H300, H304, H310, H330, H370, H372)	Environmentally toxic (H400, H410, H411)	Highly reactive with water (H260, H261)	Highly flammable (H220, H222, H224, H250)	Highly corrosive (H290, H314, H318)	Explosive (H200, H201, H202, H203, H204, H205, H240, H241, H271)	
**Polythiophene by KCTP**	**P5**	**60**	**1**	76	**1.3**	1	2	0	4	**7**	**0**	6	4	5	3	0	0	0	0	**18**
	**P10**	**60**	**1**	77	**1.3**	1	2	0	4	**7**	**0**	6	4	5	3	0	0	0	0	**18**
	**P20**	**60**	**1**	75	**1.3**	1	2	0	4	**7**	**0**	6	4	5	3	0	0	0	0	**18**
	**P30**	**65**	**1**	55	**1.8**	1	2	0	4	**7**	**0**	6	4	5	3	0	0	0	0	**18**
	**P50**	**68**	**1**	43	**2.3**	1	2	0	4	**7**	**0**	6	4	5	3	0	0	0	0	**18**

S3. Polymer characterization

S3.1. Ultraviolet photoelectron spectroscopy (UPS)

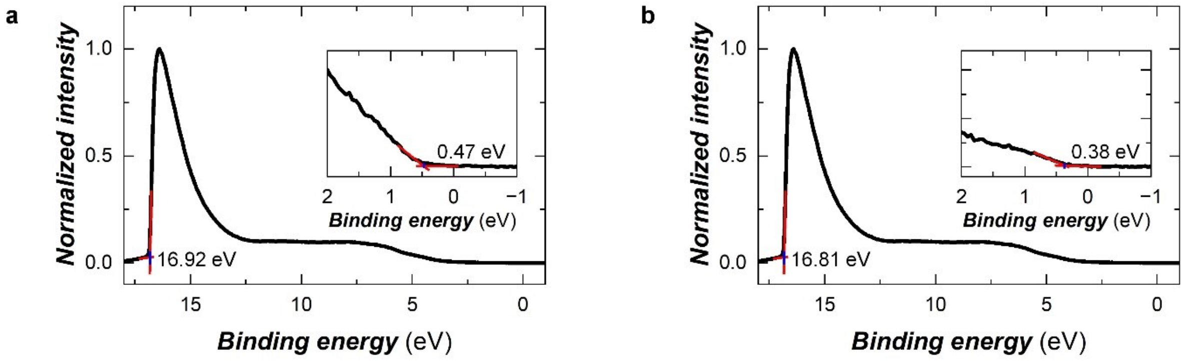

Fig. S15. UPS spectra of (a) **P12** and (b) **P35** thin films on ITO/glass substrates.

S3.2. Electrochemical characterization

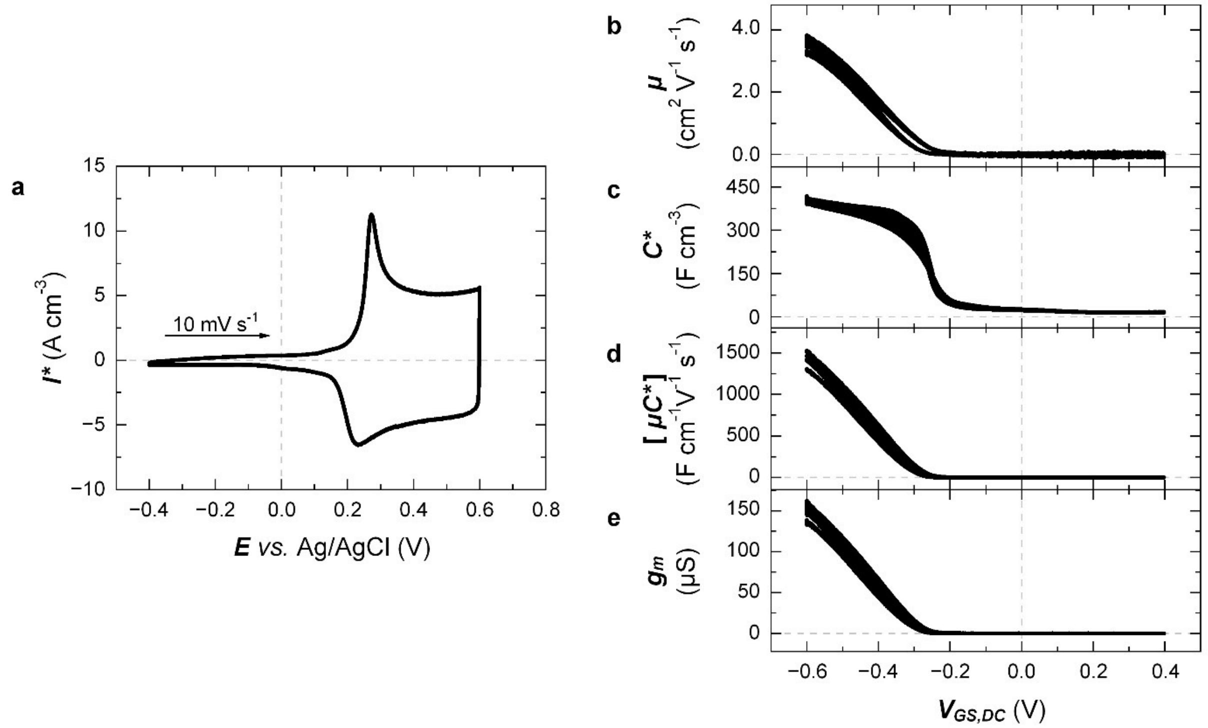

Fig. S16. Electrochemical characterization of P12. (a) Cyclic voltammetry of thin films; small signal analysis of OECTs yields (b) mobility μ, (c) volumetric capacitance C^*, (d) figure of merit $[\mu C^*]$ and (e) transconductance g_m as a function of gate voltage $V_{GS,DC}$; the material characterized was stored in air for 2 weeks after synthesis.

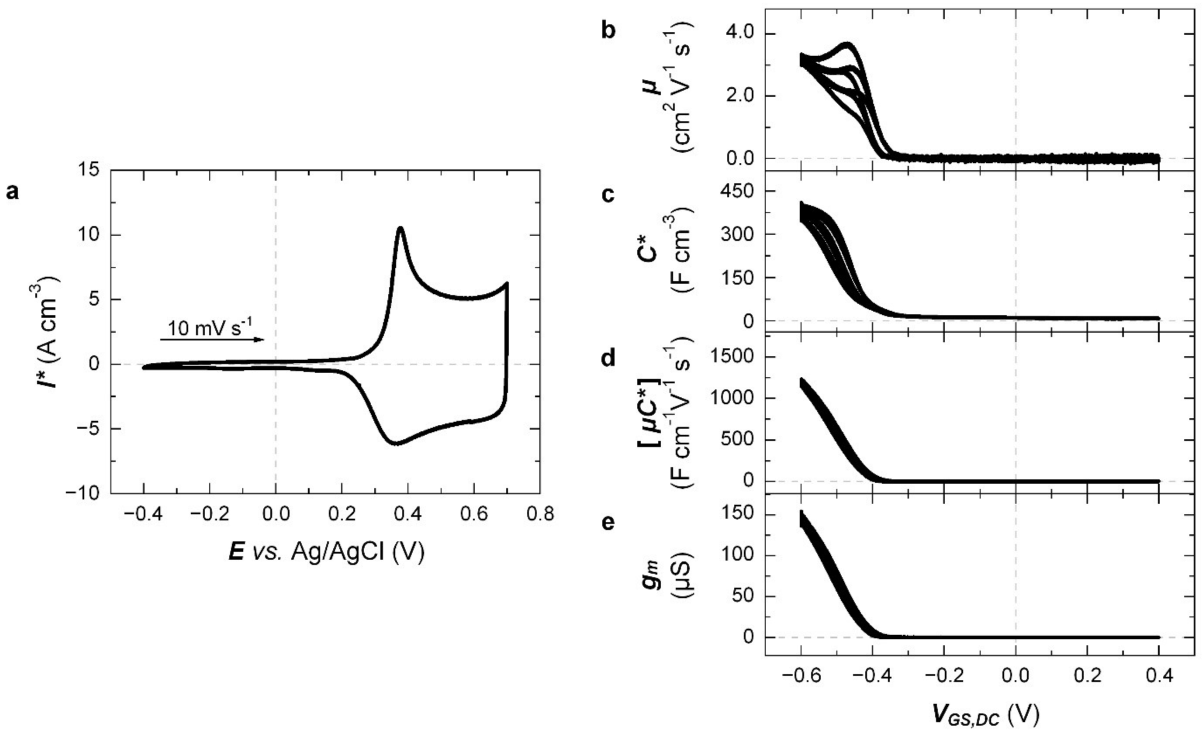

Fig. S17. Electrochemical characterization of P35. (a) Cyclic voltammetry of thin films; small signal analysis of OECTs yields (b) mobility μ, (c) volumetric capacitance C^*, (d) figure of merit $[\mu C^*]$ and (e) transconductance g_m as a function of gate voltage $V_{GS,DC}$; the material characterized was stored in air for 2 weeks after synthesis.

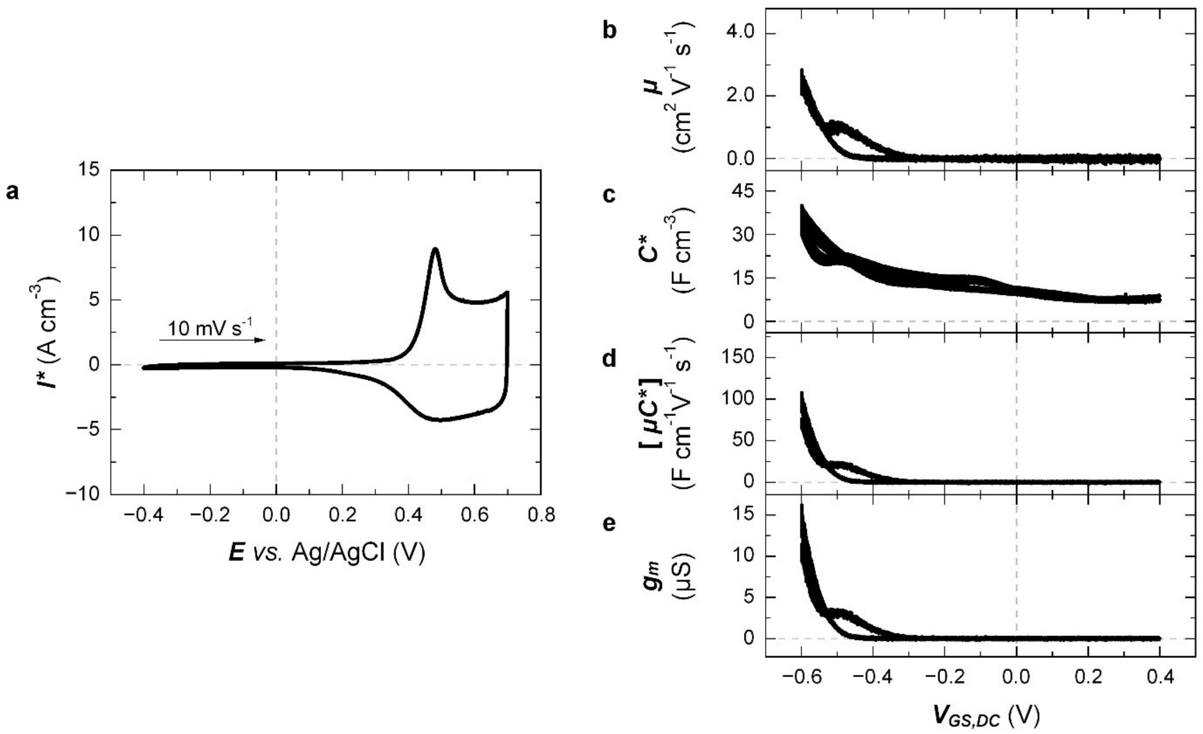

Fig. S18. Electrochemical characterization of P59. (a) Cyclic voltammetry of thin films; small signal analysis of OECTs yields (b) mobility μ, (c) volumetric capacitance C^*, (d) figure of merit $[\mu C^*]$ and (e) transconductance g_m as a function of gate voltage $V_{GS,DC}$; the material characterized was stored in air for 2 weeks after synthesis.

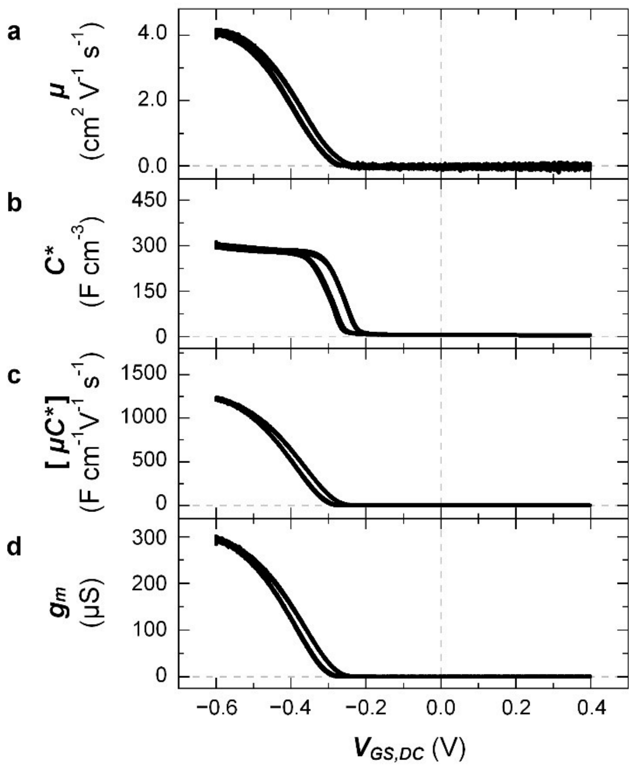

Fig. S19. OECT performance characterization of P12 stored in air for 1.5 years. Small signal analysis of OECTs yields (a) mobility μ, (b) volumetric capacitance C^*, (c) figure of merit $[\mu C^*]$ and (d) transconductance g_m as a function of gate voltage $V_{GS,DC}$.

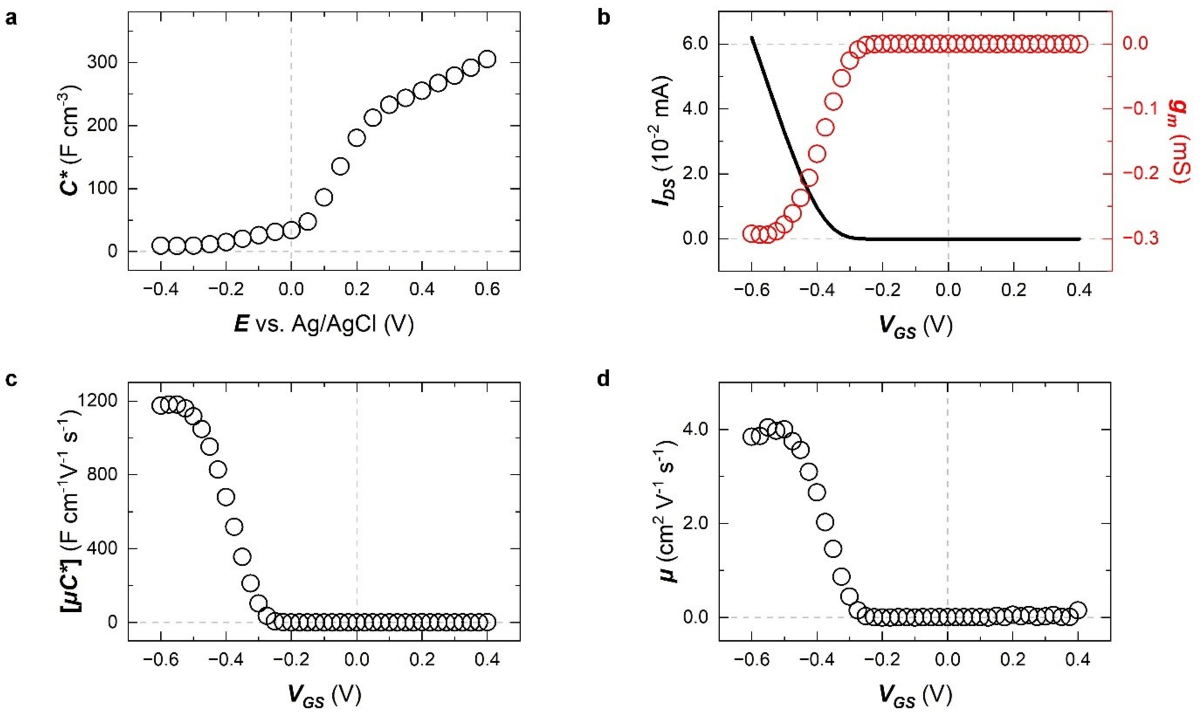

Fig. S20. OECT performance of P12 by steady-state transfer characterization. (a) Volumetric capacitance C^* from electrochemical impedance spectroscopy (EIS); (**b)** transfer curves (black) and transconductance g_m (red), which is the first derivative of drain current with respect to gate potential dI_{DS}/dV_{GS} as a function of gate potential V_{GS}; the characterization was conducted with a forward scan of V_{GS} from −0.6 V to +0.4 V in the linear regime (V_{DS} = +0.01 V); (c) figure of merit $[\mu C^*]$ obtained from transfer curves; (d) mobility μ calculated by dividing $[\mu C^*]$ by C^*; the material characterized was stored in air for 1.5 years after synthesis.

Table S4. OECT device performance by steady-state transfer curve characterization.

Maximum value of figure of merit $[\mu C^*]_{max}$ obtained from OECT transfer curves, maximum volumetric capacitance C^*_{max} determined through EIS; maximum mobility μ_{max} obtained by dividing $[\mu C^*]$ by C^*; values within brackets denote the corresponding gate potential for each value; the means and standard deviations of the $[\mu C^*]_{max}$ and μ_{max} values extracted from four channels are given; [a]from P2 in ref. [6]; [b]stored in air for 1.5 years.

Polymer	$[\mu C^*]_{max}$ (F cm^{-1} V^{-1} s^{-1})	C^*_{max} (F cm^{-3})	μ_{max} (cm^2 V^{-1} s^{-1})
p(g_3TT-T2)[a]	368 ± 9 (−0.55 V)	> 215 ± 24 (−0.6 V)	1.8 ± 0.2 (−0.5 V)
P12[b]	1180 (−0.58 V)	305 (−0.6 V)	4.0 (−0.55 V)

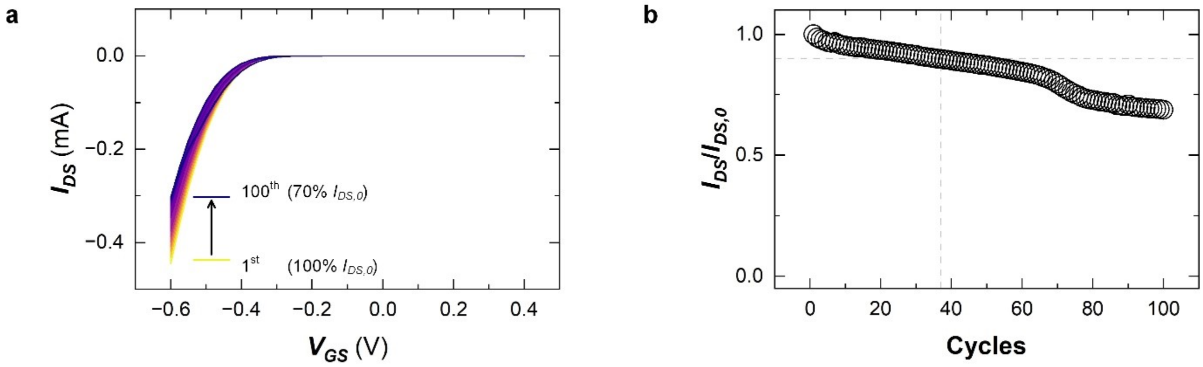

Fig. S21. OECT cycling stability of P12. (a) Transfer curves of 100 cycles with the gate potential V_{GS} varying from -0.6 V to +0.4 V at a drain potential V_{DS} = -0.6 V (yellow → blue: 1st cycle → 100th cycle); (b) relative on-current at V_{GS} = -0.6 V compared to the initial cycle $I_{DS}/I_{DS,0}$.

S3.3. Grazing incidence wide-angle X-ray scattering (GIWAXS)

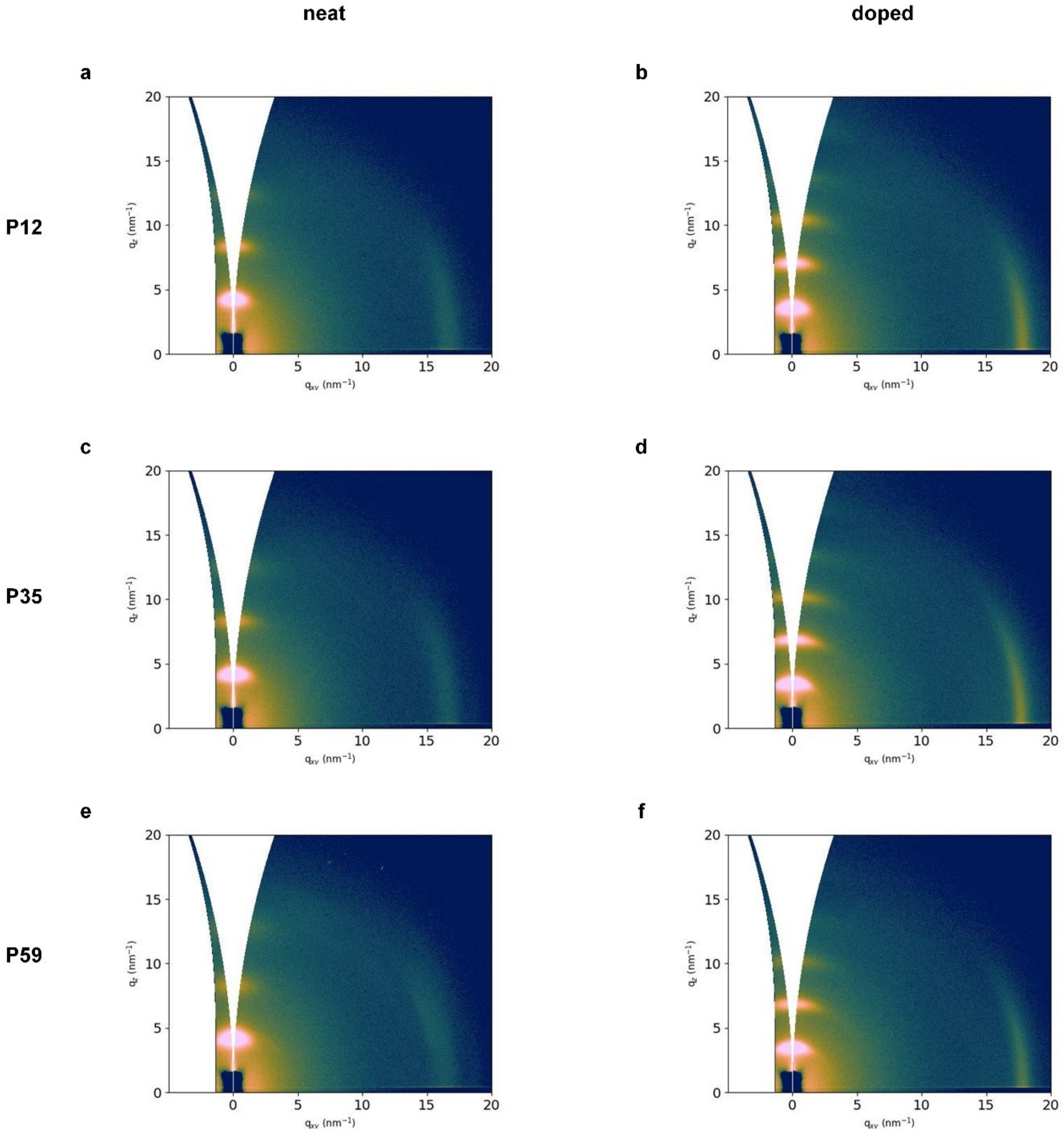

Fig. S22. GIWAXS patterns of neat and F_4TCNQ-doped thin films.

S3.4. Electrical and mechanical properties

Table S5. Electrical and mechanical properties. Elastic modulus of neat and F_4TCNQ-doped thick polymer films, E_{neat} and E_{doped}, electrical conductivity of F_4TCNQ-doped thick and thin films, σ_{thick} and σ_{thin}; σ values are obtained by measuring one sample and error bars reflect the uncertainty in resistance and thickness while in case of E the mean and standard deviation of 9 nanoindentation creep measurements of the same sample are shown.

polymer	E_{neat} (MPa)	E_{doped} (MPa)	σ_{thick} (S cm^{-1})	σ_{thin} (S cm^{-1})
P12	143 ± 9	865 ± 64	203 ± 40	390 ± 12
P35	266 ± 59	985 ± 387	408 ± 127	405 ± 4
P59	195 ± 13	736 ± 25	140 ± 27	202 ± 6

S4. References

1. O. V. Dolomanov, L. J. Bourhis, R. J. Gildea, J. A. K. Howard and H. Puschmann, OLEX2: a complete structure solution, refinement and analysis program, *J. Appl. Crystallogr.*, 2009, **42**, 339-341.

2. M. A. Viswamitra, Crystal Structure of Copper Ammonium Oxalate Dihydrate, Cu(NH4)2(C2O4)2·2H2O, *J. Chem. Phys.*, 1962, **37**, 1408-1414.

3. Y. Kim, J. Kimpel, A. Giovannitti and C. Müller, Small signal analysis for the characterization of organic electrochemical transistors, *Nat. Commun.*, 2024, **15**, 7606.

4. J. Kimpel, I. Anderson, D. Zhu, J. Kala, P. Sowinski, A. Giovannitti, L. Öhrström, J. Nelson and C. Müller, From π–π Stacking to Chain Entanglements: Single Crystals of Oligoether-Substituted Thieno[3,2-b]thiophenes, *Macromolecules*, 2026, **59**, 4612-4621.

5. J. Kimpel, Y. Kim, H. Schomaker, D. R. Hinojosa, J. Asatryan, J. Martín, R. Kroon, M. Sommer and C. Müller, Open-flask, ambient temperature direct arylation synthesis of mixed ionic-electronic conductors, *Sci. Adv.*, 2025, **11**, eadv8168.

6. J. Kimpel, Y. Kim, J. Asatryan, J. Martín, R. Kroon and C. Müller, High-mobility organic mixed conductors with a low synthetic complexity index via direct arylation polymerization, *Chem. Sci.*, 2024, **15**, 7679-7688.
